# A multifaceted lifestyle program for weight loss in overweight adults: evidence from a randomised clinical trial

**DOI:** 10.1007/s00394-025-03873-w

**Published:** 2026-01-12

**Authors:** Harsharn Gill, Chintha Lankatillake, Elena Zafiris, Christopher Pillidge

**Affiliations:** 1 School of Science, RMIT University, Melbourne, VIC Australia; 2 School of Health and Biomedical Sciences, RMIT University, Bundoora, VIC Australia

**Keywords:** Lifestyle program, Dietary fibre, Weight loss, CRP, Blood lipids, Glucose homeostasis

## Abstract

**Purpose:**

Obesity and associated metabolic disorders represent a major global health challenge. The objective of this study was to determine the efficacy of a Multifaceted Lifestyle Program (MLP, combining dietary advice and supplementation with dietary fibre, probiotics and chromium) on weight, body composition and markers of metabolic health in overweight adults.

**Methods:**

Fifty-seven overweight adults (BMI 25–29.9 kg/m^2^, aged 19–65 years) participated in an 8-week intervention trial, with 50 completing the study. Participants were randomised into three groups (A–C): A: placebo (rice powder; control); B: MLP + BioPB (an equal part blend of carrageenan, konjac and cellulose); and C: MLP + P (psyllium fibre). The primary outcome was changes in anthropometric and metabolic health indices at 4 and 8 weeks. The secondary outcome was the effect of the different fibres.

**Results:**

At week 4, participants receiving MLP + P and MLP + BioPB exhibited significant reductions (*p* < 0.001) in body weight (− 4.8 kg; and − 5.1 kg, respectively), BMI (− 1.7 kg/m^2^ and − 1.7 kg/m^2^, respectively), body fat mass (− 3.7 kg and − 4.2 kg) and visceral fat scores (− 1.9 and − 2.4). Some lean tissue mass loss was also evident. These changes were sustained for 4 weeks post-intervention. MLP + P and MLP + BioPB showed differing effects on blood lipids and inflammatory markers: reductions in cholesterol and LDL-cholesterol were greater and more persistent in the MLP + BioPB group, whereas the CRP levels (high CRP levels are linked to inflammation and metabolic disorders) were significantly reduced in the MLP + P group only. Significant improvements were also observed in the liver and kidney functions. No notable changes were observed in any parameters in the control group.

**Conclusion:**

The MLP, incorporating fibre supplementation, produced clinically significant improvements in body weight, BMI and body fat in overweight adults, offering a practical strategy for managing obesity. Importantly, the type of dietary fibre used had distinct metabolic effects, underscoring the need for tailored fibre interventions to optimise metabolic health.

**Supplementary Information:**

The online version contains supplementary material available at 10.1007/s00394-025-03873-w.

## Introduction

The prevalence of overweight and obesity has reached epidemic proportions, with 51% of the global population predicted to be living with either overweight or obesity by 2035 [1]. This has led to a significant increase in the prevalence of metabolic abnormalities (for example, abdominal obesity, hyperglycaemia and dyslipidaemia), which are linked with increased risk of cardiovascular disease (CVD), type 2 diabetes mellitus (T2DM), hypertension, chronic kidney disease, and some forms of cancer [2–5]. Excessive intake of energy-dense foods, high in fat and sugar and low in dietary fibre, and a sedentary lifestyle are linked with the rapid rise in obesity [6, 7]. Conventional intervention approaches to weight management, including either dietary advice, calorie-restricted diets, exercise, time-restricted eating (TRE), behavioural modifications, or pharmacological agents [8, 9] have shown limited long-term efficacy. Moreover, some of these interventions are associated with adverse health effects—such as nutrient deficiencies from calorie restriction and TRE [10, 11], musculoskeletal injury from inappropriate exercise regimens [12], and gastrointestinal and/or psychological side effects linked to pharmacotherapies or TRE [13, 14]. Therefore, there is an urgent need to develop multimodal interventions that target multiple pathways involved in metabolic homeostasis simultaneously. Combining conventional lifestyle modification programs (dietary and physical activity advice) with time-restricted eating protocols and supplementation with dietary fibre is one such approach that may prove more efficacious.

The effectiveness of lifestyle modification programs (which stipulate changing dietary and physical activity habits) in overweight and obese subjects has been demonstrated by several studies [15–17]. For example, the Finnish Diabetes Prevention Study and the US Diabetes Prevention Program showed that modifying dietary habits and increasing physical activity resulted in a risk reduction of almost 60% in the progression of impaired glucose tolerance to T2DM over three years [17].

Another strategy, TRE, which aligns food intake with circadian rhythm, has received considerable attention as a weight management approach in recent years. However, the results of randomised controlled TRE trials have been mixed. While some studies have reported significant reductions in body weight and improvements in markers of metabolic health in subjects following TRE, others have reported no effects [18–22]. Furthermore, the reduction in lean mass observed in participants following this eating pattern [23, 24] and the higher risk of cardiovascular deaths in at-risk subjects [25] have raised serious concerns about the benefits of this approach. However, when combined with exercise, the negative impacts of TRE on lean tissue mass are mitigated [26]. This suggests that TRE may not confer optimal benefits in isolation and should be combined with other lifestyle interventions to maximise its effectiveness. TRE is suggested to exert pleiotropic effects on multiple pathways, including improvements in hormonal regulation, restoration of normal insulin signalling pathways, promotion of metabolic switching and fat oxidation, and modulation of gut microbiota composition.

Dietary fibre supplementation represents another strategy for weight management and optimising metabolic health. While several studies have linked dietary fibre intake to improved metabolic health [27–30], some epidemiological investigations tracking fibre intake and metabolic syndrome and meta-analyses assessing the effects of specific fibre types on weight loss have reported little or no effect [31, 32]. Furthermore, systematic reviews and meta-analyses have shown high heterogeneity and publication bias in some studies and have concluded that there are insufficient high-quality studies to support the beneficial effects of dietary fibre in overweight and obese individuals [33]. Variability in dietary fibre type and source, physico-chemical properties, dose, and differences in individual responsiveness to interventions may be the reasons for these contrasting observations. This suggests that dietary fibre is inadequate as a standalone strategy to reduce weight in overweight individuals. Studies have also highlighted that supplementation of naturally occurring DF may only be efficacious when integrated with energy-restricted diets [34]. For example, chitosan was found to exhibit significant effects on weight loss, whereas glucomannan had minimal impact [35]. Similarly, PGX, a human-engineered functional dietary fibre with viscous properties, composed of konjac powder, sodium alginate and xanthan gum, was reported to be highly effective in reducing body weight independent of an energy-restricted diet [36]. Furthermore, soluble fibre, insoluble fibre and resistant starch have been reported to exert distinct effects on markers of metabolic health [37–40]. This suggests that the functional properties of dietary fibre are as important as the context of its use in supplementation, and that the development of tailored dietary fibres might be the best approach to address specific metabolic disorders for specific population groups.

Furthermore, the effectiveness of probiotics and chromium supplementation in weight management and improving metabolic health has also been the subject of several studies. Specific strains of *Lactobacillus* and *Bifidobacterium* alone or in combination with prebiotics, energy-restricted diets and/or increased physical activity have been shown to positively influence body weight and metabolic health by modulating gut microbiota composition, promoting gut barrier function and immune modulation [41–43]. Also, there is some evidence that supplementation with chromium, especially chromium picolinate, may be effective in improving glycaemic control and reducing body weight in subjects with insulin resistance and T2DM [44–46]. The strength of the evidence supporting these benefits, however, has been weak as not all studies have reported consistent effects [47–50]. The discrepancies have been attributed to differences in study design, target population groups, duration and dose of probiotics and chromium. Further research is needed to test their efficacy as stand-alone or adjuvant therapies.

Considering the complex aetiology of obesity and metabolic dysfunction, and the limitations of single interventions in obesity management, we hypothesised that a multifaceted lifestyle program (MLP) that strategically targets multiple pathways involved in metabolic homeostasis would result in significant clinical improvements in body weight and metabolic risk factors. Furthermore, we proposed that different dietary fibre formulations would exert distinct effects in overweight individuals. Therefore, the primary aim of this study was to investigate the efficacy of a MLP with two different types of dietary fibres (psyllium and BioPB) on weight, body composition and markers of metabolic health in healthy, overweight participants over 8 weeks, including a 4-week intervention, and a 4-week follow-up. BioPB (a mixture of naturally sourced konjac, carrageenan and cellulose blended in equal proportions) is a novel 3D-structured fibre with an expandable matrix developed by Biolumen Inc, USA, that has been shown in vitro to be highly effective in absorbing sugars, enhancing the production of short-chain fatty acids, and favourably modulating the composition of gut microbiota (unpublished data, Prof. R. Lustig). In terms of in vivo effects, supplementation with konjac has been shown to lower body weight and improve insulin sensitivity and lipid profiles in obese mice [51], whereas cellulose intake has been reported to improve insulin sensitivity, reduce satiety, and improve gut health [52]. On the other hand, while animal model studies suggest some benefits for carrageenan supplementation, such benefits in human trials remain to be proven [53]. Therefore, the secondary objective was to determine if different dietary fibres (psyllium and BioPB) used in combination with MLP had distinct effects on indicators of metabolic health. Effects on liver and kidney function, bowel function (stool consistency and frequency) and the health-related quality of life (HRQoL) were also assessed.

## Methods

### Ethics approval

The study protocol was approved by the RMIT Human Research Ethics Committee (RMIT HREC # 26096), and the study was carried out as per the Declaration of Helsinki guidelines. The trial was registered retrospectively at the Australia New Zealand Clinical Trials Registry (ACTRN12624001271594) and prospectively registered at the Therapeutic Goods Administration Australia (CT-2023-CTN-00822-1V3). All participants received verbal and written information about the study beforehand, and voluntary written consent was obtained from each participant before enrolment.

### Inclusion and exclusion criteria

Inclusion criteria were as follows: adults with a body mass index (BMI) of 25–29.9 kg/m^2^, apparently healthy, and able and willing to provide written consent to enrol and comply with the study guidelines.

Exclusion criteria were: a prior history of diverticulitis; allergy or intolerance to dietary fibre; use of glucose-lowering medications or use of medications known to influence body weight, lipid metabolism or gut function; use of proton pump inhibitors; history of gastrointestinal surgical interventions; coeliac disease; irritable bowel syndrome (IBS); diseases of the gallbladder; eating disorders; pregnant or breastfeeding; regular intake of laxatives in the past month and during the study; a history of eating disorders; documented chronic diseases (e.g. T2DM, cancer, liver or renal failure); currently enrolled in another study; anaemic and/or difficulty with blood draws; psychiatric disorders; weight instability in the previous 3 months; taking other contraindicated medication (i.e., antibiotics, psychotropic drugs or appetite suppressants); use of any dietary supplement that may interfere with the results of the study; and/or unable or unwilling to provide informed consent.

### Sample size

Based on previous studies [54, 55], we calculated that a minimum of 18 subjects/group will be required to detect a weight loss of 4 kg (± 2.0 kg, SD) from the baseline using an alpha (effect size) of 0.05 and a power of 0.8. Considering an attrition rate of 10%, we aimed to recruit 20 subjects/group.

### Participant recruitment

**Fig. 1:**
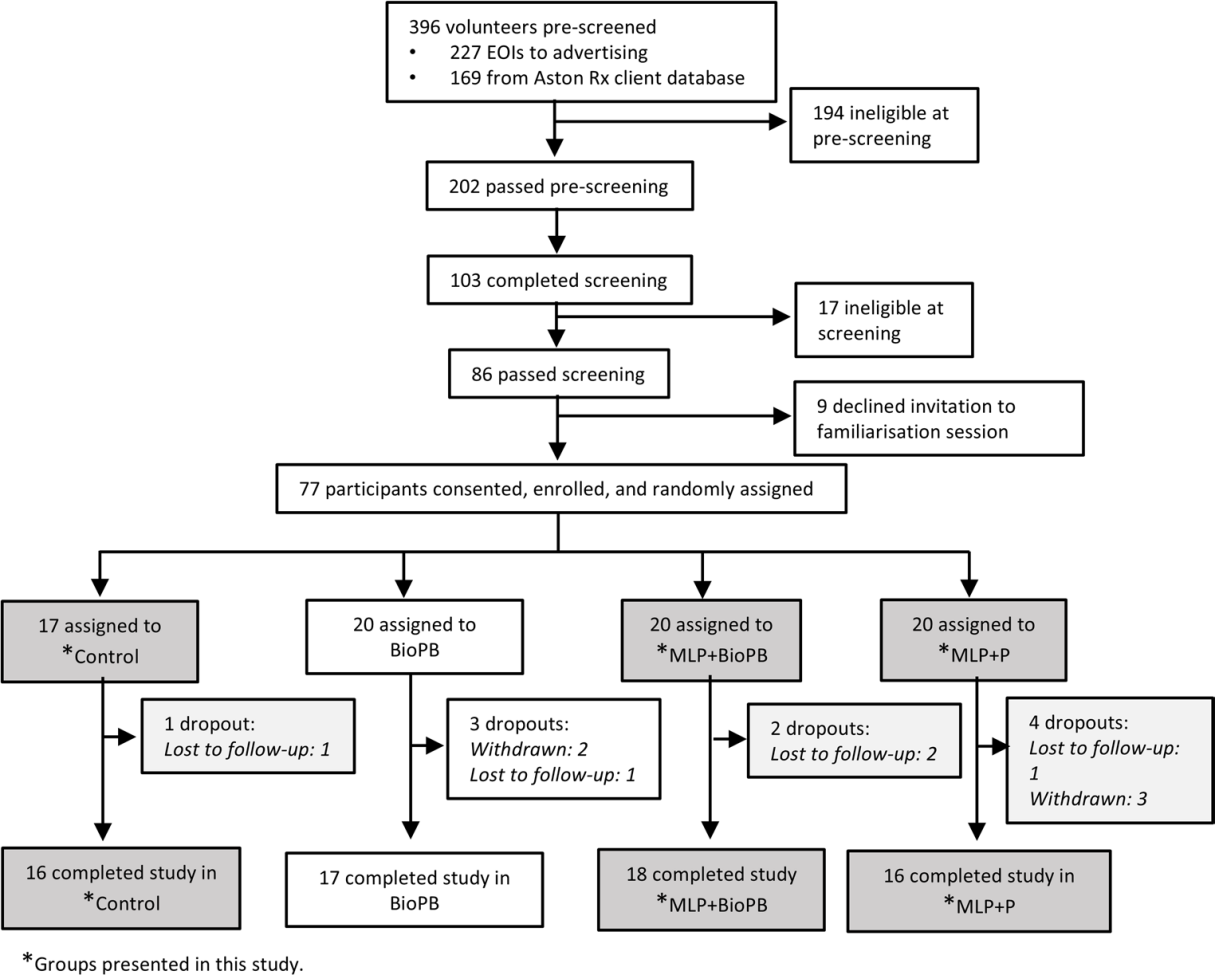
CONSORT flowchart illustrating the process for enrolment and intervention allocation

Participants were recruited between 13/04/2023 and 19/04/2023 via public advertising and from AstonRx’s client database, which contains over 100,000 contacts who have expressed interest in its weight loss programs (Metabolic Reboot Program) over the past five years. In total, 227 volunteers responded to advertising and a further 169 were pre-screened from the Aston Rx client database **(**Fig. 1**)**. Out of these 396 subjects, 86 were found to be eligible. The most common reasons for ineligibility were BMI > 30, followed by volunteers having been diagnosed with an excluded medical condition and/or being on a contraindicated medication. A further nine subjects declined the invitation to participate, leaving 77 subjects who enrolled in the study. Of these, five withdrew after enrolment, and five were lost to follow-up, leaving 67 participants who completed the study (Fig. 1).

Here, we present results from three (n = 50) of the four intervention groups (n = 67). A flowchart summarising the process for enrolment and intervention allocation is shown in Fig. 1. The study protocol is available in Supplementary Information.

### Trial design and intervention

The trial was designed as an 8-week (4-week intervention [30/04/2023–03/06/2023] and 4-week follow-up [04/06/2023–01/07/2023]), randomised, single-blind (with unblinded lifestyle modification advice), placebo-controlled, parallel-design study and was conducted in Melbourne, Australia between April 13, 2023, and July 03, 2023. Participants were randomly assigned to one of the four intervention groups (Fig. 1). All supplements provided to subjects were identical in appearance and packaging to maintain blinding. Randomisation was via stratified, permuted block randomisation using the random number generator in Microsoft Excel, with a 1:1 allocation ratio, and stratification according to gender, BMI, and whether participants had undergone an appendectomy, a cholecystectomy or neither. Random sequence generation and allocation were carried out by CL. The identity of the groups (i.e. intervention type) was unknown to CL at the time of randomisation and allocation. Participants were enrolled by HG, CP, and a small number of non-author support staff.

This study reports the results from three groups only. Randomisation was as follows: Group A, the control group received a placebo (rice powder, n = 17); Group B, received the MLP with BioPB (MLP + BioPB, n = 20); and Group C, received MLP with psyllium dietary fibre (MLP + P, n = 20). The number of participants who completed the trial for each of these groups was: A, 16; B, 18; C, 16 (Fig. 1).

The intervention period was four weeks. During the intervention period, participants consumed either 4.2 g of BioPB, psyllium or the placebo twice daily, sprinkled over their meals. BioPB comprised equal proportions of carrageenan, konjac and cellulose (hence comprising about 60% soluble fibre and 40% insoluble fibre). After the cessation of the intervention period, participants were followed up for an additional four weeks.

To facilitate adherence, participants were provided with clear written and verbal instructions on how and when to take the fibre supplements. Adherence to the fibre supplement protocol was monitored using an Investigational Product (IP) accountability log. Participants were instructed to return any unused sachets at Visit 2 (Week 4), and the number of returned products was recorded to assess compliance.

#### Multifaceted lifestyle program (MLP)

The MLP chosen for this study was a 28-day customised online lifestyle modification program that provided participants with access to a web-based interactive dashboard (accessible via browser), which delivered education, a detailed daily meal plan with controlled portion sizes (incorporating diverse fibrous plant foods) based on an individual’s lean body weight, and on-demand exercise videos (https://www.astonrx.com). It also instructed participants to follow time-restricted eating (TRE—circadian) and avoid consuming alcohol, sugar, refined carbohydrates or artificial sweeteners. The program commenced with 3 meals a day, with a minimum 5-h fast between meals and a minimum 12-h overnight fast. Participants were encouraged to finish eating during daylight hours and at least 3–4 h before sleep. From Day 10 onwards, participants were advised, if desired, to opt for 2 meals a day on any day they wished. The program also included supplementation with probiotics (UltraFlora Restore, Metagenics Ltd Australia; https://www.metagenics.com.au, which contains *Lactobacillus acidophilus* [NCFM^®^], *Bifidobacterium lactis* [Bi-07], and *Lactobacillus rhamnosus* [LGG^®^]), Bio-Chromium Plus (Blackmores Limited, Australia; https://www.bioceuticals.com.au/products/bio-chromium-plus).

A separate fibre group (BioPB fibre alone, n = 17 completers) was also included in the study. However, the results were excluded from the main manuscript as the fibre supplementation alone did not significantly affect any of the anthropometric measures (the study’s primary outcome); the data are instead presented in Fig. S4.

### Outcomes

Primary outcomes included changes in anthropometric measures and body composition from the baseline, at Weeks 4 and 8. Secondary outcomes included changes in biomarkers of metabolic health.

### Study visits

Participants attended an initial screening and measurement session (Visit 1, Week 0/Baseline), and two additional measurement sessions (Visit 2, Week 4 and Visit 3, Week 8). At Visit 1, volunteer eligibility to participate was confirmed. Eligible volunteers consented and enrolled in the study, and participants underwent baseline anthropometric and body composition measurements. Anthropometric and body composition measurements were also conducted at visits 2 (Week 4) and 3 (Week 8).

### Blood collection and analysis

Blood samples after overnight fasting were collected by a professional phlebotomist at a conveniently located clinic (Melbourne Pathology; pathology collector) and forwarded to NutriPATH (Melbourne) (https://nutripath.com.au/) for analysis of key metabolic health biomarkers (serum was used for measuring the levels of lipids, glucose, insulin, hs-CRP, liver and kidney enzymes). Blood sampling was performed at Week 0 (baseline/preintervention), Week 4 (post-intervention), and Week 8 (end of the follow-up period). Participants also completed questionnaires/surveys at each of these time points.

#### Homeostatic Model Assessment for Insulin Resistance (HOMA-IR) score calculation

HOMA-IR values were calculated by using the following formula [56]:

*HOMA–IR* = Fasting Glucose (mmol/L) × Fasting Insulin (µU/mL)/22.5

### Anthropometric and body composition measurements

Anthropometric, body composition and blood pressure measurements were taken at Weeks 0, 4 and 8. Standing weight and height measurements were recorded using a 570 Biometric Composition Analyser with a BSM370 stadiometer (InBody Body Composition Analysers Pty Ltd, Queensland, Australia; https://au.inbody.com) with subjects wearing light clothing and no shoes. BMI, body circumference, waist-to-hip ratio, and body composition measurements (percentage body fat [PBF], visceral fat level, skeletal muscle mass [SMM]) were analysed using a bioelectrical impedance body composition analyser (InBody 570^®^, InBody Body Composition Analysers Pty Ltd, Queensland, Australia). Blood pressure and pulse were measured at each visit using an automated, electronic blood pressure monitor (BPBIO 320, InBody Body Composition Analysers Pty Ltd, Queensland, Australia). The stadiometer and blood pressure monitor were integrated with the InBody 570^®^ body composition analyser.

### Surveys and questionnaires

The following surveys/questionnaires were administered as per the timelines provided: RAND 36 Item Short Form Health Survey, Gastrointestinal Symptoms and Stool Output Questionnaire—at Weeks 0, 4, and 8, and Reported Side Effects Questionnaires at Weeks 1, 2, 3, 4, 5, and 8. The survey questionnaires are provided in the supplementary data (Supplementary questionnaires S1–S3, Supplementary Material).

The RAND 36-Item Short Form Health Survey (SF-36), developed by the RAND Corporation, is a widely used, validated instrument for assessing health-related quality of life across multiple domains [57]. The survey consists of 36 items, each rated on a Likert scale, with response options varying from three to six points depending on the question. It has demonstrated strong reliability (Cronbach’s alpha > 0.85) and validity in diverse populations and is considered a reliable tool for measuring general health status in clinical and research settings [58–60].

The Gastrointestinal Symptoms and Stool Output Questionnaire consisted of the Gastrointestinal Symptoms Rating Scale (GSRS) for monitoring common gastrointestinal symptoms and the Bristol Stool Form Scale for assessing stool type [61]. The GSRS, developed by Svedlund et al. [62], is a validated instrument designed to assess the severity of 15 common gastrointestinal symptoms, such as abdominal pain, heartburn, and bloating. Each symptom is rated on a 4-point Likert scale based on intensity and frequency. The GSRS has demonstrated strong reliability and validity, with high interrater and internal consistency reliability across different populations [63]. The Bristol Stool Form Scale is a validated, widely used tool that classifies human stool into seven distinct types based on appearance and consistency, ranging from hard lumps (type 1) to entirely liquid (type 7). It has demonstrated reliability and validity in both clinical and research settings [64].

The Reported Side Effects Questionnaire was developed by our research team based on common side effects expected from dietary fibre supplementation. The survey included 7 symptoms (nausea, stomach pain, abdominal discomfort/cramps, bloating, diarrhoea, flatulence, and constipation) rated using a 6-point Likert scale based on frequency. This approach ensured that the questionnaire was tailored to capture the most relevant and anticipated symptoms associated with increased dietary fibre intake in our study population.

### Dietary intake and physical activity monitoring

Participants completed 3-day food diaries at Weeks 0 (pre-intervention), 4 (post-intervention), and 8 (4 weeks post-intervention). The food consumption data were categorised into 13 Dietary Categories (DCs; adapted from the WHO/FAO GIFT [Food and Agriculture Organisation/World Health Organisation Global Individual Food consumption data Tool] [65] to track the presence or absence of key food groups at each mealtime. The Dietary Categories were coffee, alcohol, sugar-based beverages, sweet and savoury snacks, cereals, starchy vegetables, fruit, milk and milk products, legumes, nuts and seeds, vegetables (non-starchy), meat and eggs, and fish and seafood.

For each participant, at each time point (Week 0, 4 and 8) for each meal (breakfast, lunch, dinner, snacks, drinks) each DC was coded as 1 if present and 0 if absent. Composite meals were assessed using a conservative, evidence-based approach. Only explicitly reported ingredients were coded. We then calculated the percentage of meals including each Dietary Category per group per timepoint:

Percentage of meals including each food group = (Number of meals including food group/Total meals in Group at Timepoint) * 100.

This approach standardised comparisons across groups and time points, accounting for variations in the number of meals within groups. Physical activity was monitored based on 3-day lifestyle diaries completed by participants, and the duration of their activity was calculated as detailed in Supplementary Methods.

#### Data analysis

All data were analysed using GraphPad Prism (Prism 10 for Windows 64-bit, Version 10.4.2 [633]). The anthropometric and body composition data, blood biochemistry, blood pressure and pulse data were assessed for the assumption of normality and homoscedasticity using the Shapiro–Wilk test and Levene’s test, respectively. The changes in primary and secondary outcomes were analysed as within-group changes from baseline, calculated as differences at Weeks 4 and Week 8 compared to Week 0 (i.e., Week 4 minus Week 0 and Week 8 minus Week 0), and the differences across timepoints (baseline, week 4, and week 8) were assessed using the Friedman test, a non-parametric repeated measures one-way ANOVA. For this, the changes at baseline were set to zero and used as the reference comparison point. Where significant differences were detected, Dunn’s multiple comparisons test was used to compare changes at week 4 and week 8 to baseline. Data are presented as mean change ± standard error of the mean (SEM), unless otherwise stated. Statistical significance was accepted at* p* < 0.05.

#### Data exclusions

hs-CRP data for a small number of participants who became unwell (all with coronavirus disease 2019 [COVID-19] infections, except for one who had another illness unrelated to the study) and had high hs-CRP values were excluded from the statistical analysis. This involved excluding one participant from the Control group and one from the MLP + P group at Week 8.

There were no changes to the methods or outcomes after trial commencement. The trial concluded upon completion of data collection at the end of the follow-up period.

## Results

### Baseline characteristics

Of the 57 participants randomised into three groups, seven withdrew before the commencement of intervention or were lost to follow-up. Fifty participants completed the study (Fig. 1, Table 1). The mean age of participants was 48.9 years. Thirty-nine were female, and eleven were male. The mean height, weight, and BMI were 169.0 cm, 81.4 kg, and 28.3 kg/m^2^, respectively.

**Table 1:** Baseline characteristics of study participants

	Control	MLP + BioPB	MLP + P
*n*	16	18	17
*Sociodemographic characteristics*			
Age (years)	47.9 ± 9.2	49.8 ± 7.3	48.6 ± 5.6
Males	5	4	3
Females	11	14	14
Regularly consume alcohol	12	12	12
Current smokers	0	1	0
Ex-smokers	6	5	6
Have never smoked	10	12	11
*Anthropometric characteristics*			
Height (cm)	169.3 ± 10.9	169.6 ± 8.2	168.8 ± 6.1
Weight (kg)	82.2 ± 12.7	81.6 ± 10.8	80.3 ± 8.9
BMI (kg/m^2^)	28.5 ± 1.9	28.3 ± 1.8	28.1 ± 1.9
*Body composition characteristics*			
Waist-Hip Ratio	0.96 ± 0.07	0.97 ± 0.06	0.97 ± 0.05
Waist-Height Ratio	0.58	0.59	0.59
Visceral Fat Level	12 ± 3	13 ± 3	13 ± 3
Body Fat Mass (kg)	26.8 ± 4.7	27.2 ± 5.5	28.0 ± 4.3
Percentage Body Fat (%)	33.3 ± 7.1	33.4 ± 6.0	35.0 ± 4.5
Skeletal Muscle Mass (kg)	30.9 ± 8.2	30.3 ± 6.1	28.9 ± 4.5
*Blood pressure & pulse*			
Systolic (mmHg)	133 ± 13	130 ± 14	133 ± 17
Diastolic (mmHg)	80 ± 8	75 ± 11	75 ± 11
Pulse (bmp)	71 ± 10	72 ± 9	74 ± 11
*Biochemical parameters*			
Cholesterol (mmol/L)	5.9 ± 0.9	6.3 ± 1.0	5.7 ± 0.8
Triglycerides (mmol/L)	1.0 ± 0.4	1.0 ± 0.5	1.0 ± 0.4
HDL (high-density lipoprotein; mmol/L)	1.9 ± 0.4	2.0 ± 0.5	1.8 ± 0.3
LDL (low-density lipoprotein; mmol/L)	3.6 ± 0.7	3.9 ± 0.9	3.5 ± 0.7
LDL/HDL ratio	2.0 ± 0.5	2.1 ± 0.7	2.0 ± 0.5
Triglyceride/HDL ratio	0.5 ± 0.2	0.5 ± 0.4	0.6 ± 0.3
Fasting glucose (mmol/L)	5.3 ± 0.7	5.3 ± 0.5	5.5 ± 0.7
Fasting insulin (mU/L)	6.1 ± 2.6	6.9 ± 4.0	7.7 ± 5.1
HOMA IR score	1.4 ± 0.6	1.6 ± 1.0	1.9 ± 1.4
Creatinine (serum; µmol/L)	67.4 ± 14.3	72.2 ± 9.5	68.4 ± 10.2
Bilirubin (µmol/L)	6.7 ± 3.3	8.6 ± 4.6	6.1 ± 3.0
Albumin (serum; g/L)	40.1 ± 2.2	41.8 ± 2.1	41.0 ± 2.1
Total protein (serum; g/L)	69.2 ± 4.5	70.2 ± 3.5	68.9 ± 3.9
ALP (units/L)	73.4 ± 23.1	64.3 ± 14.9	66.2 ± 16.9
ALT (units/L)	26.4 ± 11.3	26.9 ± 13.5	20.8 ± 5.0
AST (units/L)	27.0 ± 5.9	26.3 ± 5.4	23.1 ± 4.0
GGT (units/L)	12.5 ± 6.8	15.1 ± 18.7	10.8 ± 11.6
High sensitivity CRP (mg/L)	1.80 ± 1.76	1.55 ± 2.09	1.72 ± 1.48

Values represent the mean ± standard deviation (SD) for the Control, MLP + BioPB (multifaceted lifestyle program with BioPB dietary fibre), and MLP + P (MLP with psyllium) groups

### Dietary intake and physical activity

An inherent problem with self-reported food diaries is the inability to capture data of sufficient quality [66]. The dietary information provided by the participants was inconsistent and lacked sufficient information for quantification using software such as Foodworks (https://foodworks.online/). While participants failed to consistently note how much food they consumed, they consistently reported on what was consumed. Therefore, we adopted a semi-quantitative analysis of the dietary data as described in the Methods section.

**Fig. 2:**
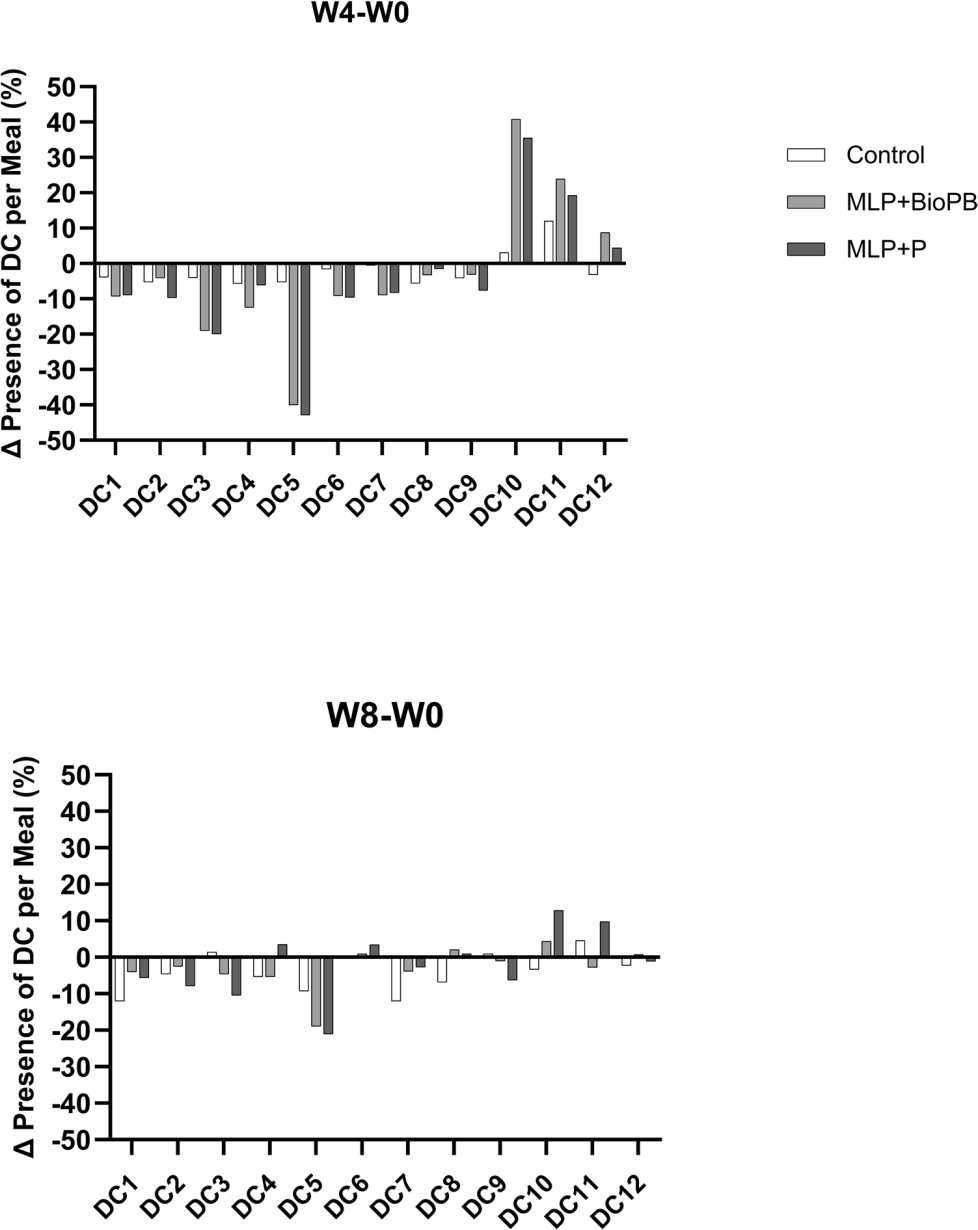
Group-level changes in dietary habits—expressed as the change in percentage of meals including each dietary category—from baseline (Week 0) to Week 4 across study arms. Each bar represents the percentage-point difference in presence per meal (% at Week 4 minus % at Week 0) for Control (black), MLP + BioPB (red) and MLP + P (green). This visualisation provides a qualitative indication of dietary shift across intervention groups; no statistical testing was performed due to the descriptive nature of the analysis. Dietary Categories are as follows: DC1 Alcohol; DC2 Sugar-Based Beverages; DC3 Sweets & Savory Snacks; DC4 Starchy Vegetables; DC5 Fruit; DC6 Cereals; DC 7 Milk & Milk Products; DC8 Vegetables (non-starchy); DC9 Nuts & Seeds; DC10 Legumes; DC11 Meat & Meat Products; DC 12 Fish & Seafood

During the intervention, subjects’ cereal intake dropped significantly while non-starchy vegetables increased substantially across the MLP + P and MLP + BioPB groups and remained lower post-intervention compared to baseline (Fig. 2). Meat and egg consumption also increased during the intervention, particularly in MLP + P and MLP + BioPB groups and stabilised at a level similar to the pre-intervention levels by Week 8. Alcohol, sugary beverages, and sweet and savoury snacks saw notable reductions across all groups during the intervention, with a slight rebound afterwards; however, levels remained lower than baseline, particularly in MLP + P and MLP + BioPB groups. Meanwhile, fruit, milk and milk products, legumes, nuts and seeds showed less dramatic changes, with only minor fluctuations across different time points and groups. These results highlight the intervention’s positive impact on reducing the consumption of high and simple carbohydrate-accessible foods, such as cereals, while increasing the intake of foods with lower carbohydrate accessibility, such as non-starchy vegetables, meat and eggs.

The levels of physical activity, based on participants’ lifestyle diaries, are presented in Supplementary Fig. S1. Participants in both MLP + P and MLP + BioPB groups showed an increase in their calculated physical activity determined at Week 4, and this was maintained until Week 8 for the MLP + P group only; however, this increase was not statistically significant.

### Anthropometry and body composition

**Fig. 3:**
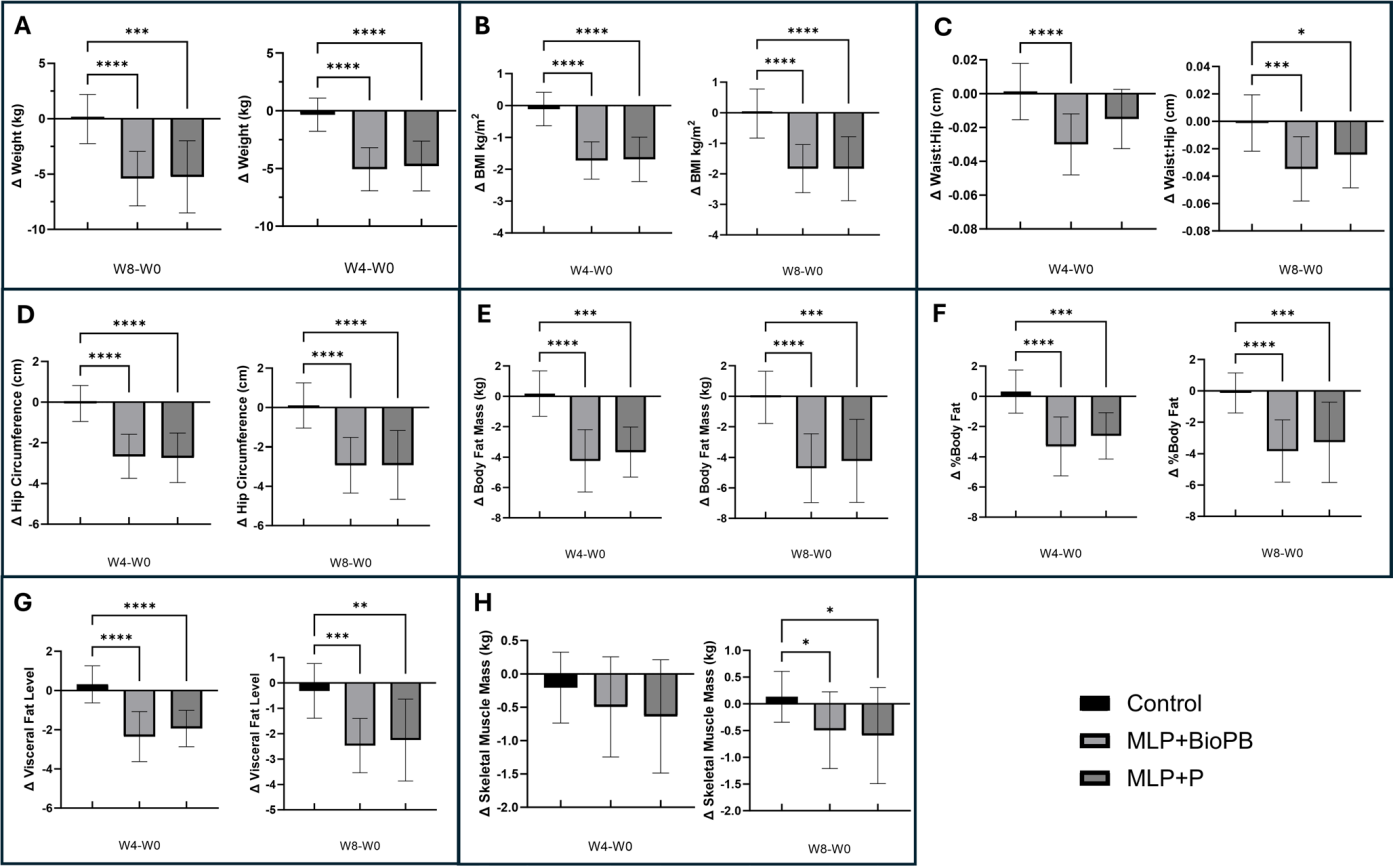
Change from baseline in key anthropometric and body composition indices at Weeks 4 and 8. This figure illustrates the changes from baseline in anthropometric indices and body composition at Week 4 and Week 8 in Control (n = 16), MLP + BioPB (n = 18), and MLP + P (n = 16) groups. Data represent changes from baseline to the end of intervention (W4-W0), and 4 weeks post-intervention follow-up (W8-W0). Panels represent the following: **A** Difference in Weight (kg), **B** Difference in Body Mass Index (kg/m^2^), **C** Difference in Waist to Hip ratio (cm), **D** Difference in Hip circumference (cm), **E** Difference in Body Fat Mass (kg), **F** Difference in Percentage of Body Fat, **G** Change in Visceral Fat Level, and **H** Change in Skeletal Muscle Mass. Statistical significance was determined using the Friedman test, a non-parametric repeated measures one-way ANOVA followed by Dunn’s multiple comparisons test, GraphPad Prism Version 10.4.2. Differences were considered significant if *p* < 0.05. ^†^The InBody 570 Biometric Composition Analyser, paired with BSM370 stadiometer, measures visceral fat and presents the result as a scaled value from 1 to 20 corresponding to visceral fat area. For instance, a score of 20 equates to 200 cm^2^ of visceral fat

Compared to the baseline data, the mean body weight of subjects in the MLP + P group decreased by 4.8 kg (95% CI − 5.9 to − 3.6 kg, *p* = 0.0002; − 5.9%, 95% CI − 7.2 to − 4.6%) at week 4 and by 5.3 kg (95% CI − 7.0 to − 3.5 kg, *p* < 0.0001; − 6.5%, 95% CI − 8.5 to − 4.5%), at week 8 (Fig. 3, Table S1). The participants in the MLP + BioPB group also exhibited significant weight reductions of 5.1 kg (95% CI − 6.0 to 4.1, *p* = 0.0002; − 6.1%, 95% CI − 7.2 to 5.1%) and 5.4 kg (95% CI − 6.7 to 4.1, *p* < 0.0001, − 6.5%, 95% CI − 7.9 to − 5.1%) at weeks 4 and 8, respectively (Fig. 3, Table S1). In contrast, the mean body weight of subjects in the control group remained unchanged. Compared to the control group, MLP + BioPB and MLP + P both exhibited a significant difference in body weight at Weeks 4 and 8 (*p* < 0.0001 at each time point).

Similarly, there was a significant reduction in mean BMI in the MLP + P and MLP + BioPB groups at Weeks 4 and 8 (Fig. 3, Table S1). The waist-to-hip ratio was significantly reduced in the MLP + BioBP group at Weeks 4 (− 0.03, 95% CI − 0.04 to − 0.02, *p* = 0.0004; − 3.06%, 95% CI − 3.99 to − 2.12%), and 8 (− 0.03, 95% CI − 0.05 to − 0.02, *p* = 0.0001; − 3.57%, 95% CI − 4.82 to − 2.31%). Significant changes in waist-to-hip ratio in the MLP + P group were only observed at Week 8, but not at Week 4. Similarly, there was a significant reduction in waist-to-height ratio in the MLP + BioPB group at Weeks 4 (− 5.66%, 95% CI − 6.8 to − 4.5%, *p* = 0.0002) and 8 (− 6.18%, 95% CI − 7.62 to − 4.74%, *p* < 0.0001). The MLP + P group also exhibited significant reductions in the waist-to-height ratio at Weeks 4 (− 4.20%, 95% CI − 5.37 to − 3.0%, *p* = 0.0011) and 8 (− 5.32%, 95% CI − 7.37 to − 3.27%, *p* = 0.0001).

For the MLP + BioPB and MLP + P groups, significant reductions were also observed for neck, chest, waist, hip, right arm, left arm, right thigh, and left thigh circumferences at Weeks 4 and 8 (Fig. 3, Table S1). However, there was no change in these measurements in the control group.

Body composition analysis (Fig. 3, Table S1) revealed that the total body fat mass (− 16.2%, 95% CI − 20.7 to − 11.6%, *p* < 0.0004), percentage body fat (− 10.7% 95% CI − 14.9 to − 6.6%, *p* < 0.0001), and visceral fat level (− 19.1%, 95% CI − 24.3 to − 13.8%, *p* < 0.0001) were significantly reduced in the MLP + BioPB group at Week 4. These changes were sustained until Week 8 (total body fat mass − 17.9% 95% CI − 22.7 to − 13.1%, *p* < 0.0001; percentage body fat − 12.2%, 95% CI − 16.2 to − 8.3%, *p* < 0.0001; and visceral fat level − 20.3%, 95% CI − 24.7 to − 15.9%, *p* < *0.0001*). The MLP + P group also demonstrated smaller yet significant reductions in these measurements at Week 4 (total body fat mass − 13.0%, 95% CI − 16.1 to − 9.9%, *p* < 0.0008; percentage body fat − 7.6%, 95% CI − 10.2 to − 5.0%, *p* = *0.0016*; and visceral fat level − 14.9%, 95% CI − 18.8 to − 11.0%, *p* = *0.0008)* and Week 8 (total body fat mass − 15.1%, 95% CI − 20.6 to − 9.7%, *p* = 0.0002; percentage body fat − 9.5%, 95% CI − 13.7 to − 5.3%, *p* < 0.0001; and visceral fat level − 17.4%, 95% CI 24.4 to − 10.4%, *p* <  = 0.002). There were no significant differences in the above measurements in the Control. Skeletal muscle mass, soft lean mass, fat-free mass and total body water were also significantly reduced in the MLP + BioPB group at both Weeks 4 and 8, whereas in the MLP + P group, reductions were only observed at Week 4 (Fig. 3, Table S1).

#### Blood lipids

There was a significant reduction in the levels of cholesterol (*p* < 0.0001), non-HDL cholesterol (*p* < 0.0001), LDL (*p* < 0.0001) and HDL (*p* = 0.004) in the MLP + BioPB group at Week 4 compared to baseline (Table 2). These changes were sustained until Week 8 in cholesterol (*p* = 0.0071, non-HDL cholesterol (*p* = 0.0202) and LDL (*p* = 0.0202). Significant reductions (*p* < 0.05) in cholesterol and LDL, but of a lower magnitude than MLP + BioPB*,* were also observed in the MLP + P group at Week 4 but not at Week 8. Levels of triglycerides remained unchanged in all groups.

**Table 2:** Cardiometabolic health biomarkers at Weeks 0 (preintervention), 4 (post-intervention) and 8 (4 weeks after the intervention had ceased)

	Mean ± (SD)			Mean Difference from Baseline (95% CI)			
	Week 0	Week 4	Week 8	Week 4		Week 8	
				Mean Difference	*p* value	Mean Difference	*p* value
*Cholesterol (mmol/L)*							
Control	5.96 (0.97)	5.57 (0.94)	5.71 (0.88)	− 0.3929 (− 0.7329, − 0.0528)	0.0280	− 0.2571 (− 0.6339, 0.1196)	ns
MLP + BioPB	6.2 (1.01)	5.27 (0.64)	5.68 (1.04)	− 0.9294 (− 1.270, − 0.5885)	< 0.0001	− 0.5176 (− 0.7427, − 0.2926)	0.0071
MLP + P	5.74 (0.83)	5.24 (1.07)	5.54 (0.99)	− 0.5000 (− 0.8398, − 0.1602)	0.0431	− 0.2000 (− 0.5464, 0.1464)	ns
*Triglycerides (mmol/L)*							
Control	0.98 (0.44)	0.81 (0.27)	1.03 (0.47)	− 0.1643 (− 0.3714, 0.04279)	ns	0.0500 (− 0.1364, 0.2364)	ns
MLP + BioPB	1.00 (0.58)	0.94 (0.37)	0.95 (0.55)	− 0.0588 (− 0.2370, 0.1193)	ns	− 0.0471 (− 0.1915, 0.09736)	ns
MLP + P	0.98 (0.45)	0.89 (0.32)	0.87 (0.26)	− 0.0875 (− 0.2829, 0.1079)	ns	− 0.1125 (− 0.2692, 0.0442)	ns
*HDL (mmol/L)*							
Control	1.86 (0.45)	1.80 (0.43)	1.75 (0.43)	− 0.0571 (− 0.1720, 0.0577)	ns	− 0.1071 (− 0.2071, − 0.0072)	ns
MLP + BioPB	1.97 (0.49)	1.75 (0.42)	1.79 (0.45)	− 0.2176 (− 0.3465, − 0.08881)	0.0040	− 0.1706 (− 0.2866, − 0.05453)	0.0412
MLP + P	1.81 (0.34)	1.63 (0.33)	1.71 (0.42)	− 0.1813 (− 0.3284, − 0.0341)	ns	− 0.1000 (− 0.2593, 0.0593)	ns
*Non-HDL cholesterol (mmol/L)*							
Control	4.1 (0.87)	3.78 (0.64)	3.96 (0.64)	− 0.3236 (− 0.6090, − 0.0381)	0.0026	− 0.1450 (− 0.4555, 0.1655)	ns
MLP + BioPB	4.24 (1.13)	3.54 (0.80)	3.88 (1.10)	− 0.7000 (− 1.033, − 0.3674)	< 0.0001	− 0.3594 (− 0.5249, − 0.1939)	0.0202
MLP + P	3.93 (0.79)	3.62 (0.92)	3.83 (0.86)	− 0.3094 (− 0.5941, − 0.0246)	ns	− 0.1000 (− 0.3920, 0.1920)	ns
*LDL (mmol/L)*							
Control	3.69 (0.75)	3.41 (0.64)	3.51 (0.58)	− 0.2714 (− 0.5781, 0.03529)	ns	− 0.1786 (− 0.4901, 0.1329)	ns
MLP + BioPB	3.8 (0.95)	3.14 (0.70)	3.47 (0.96)	− 0.6647 (− 0.9656, − 0.3638)	< 0.0001	− 0.3294 (− 0.4948, − 0.1640)	0.0202
MLP + P	3.49 (0.74)	3.21 (0.87)	3.45 (0.81)	− 0.2750 (− 0.5328, − 0.0172)	0.0431	− 0.0375 (− 0.3451, 0.2701)	ns
*Cholesterol/HDL ratio*							
Control	3.3 (0.62)	3.19 (0.48)	3.34 (0.56)	− 0.1071 (− 0.2648, 0.0505)	ns	0.0357 (− 0.0956, 0.1670)	ns
MLP + BioPB	3.31 (0.89)	3.20 (0.90)	3.29 (0.89)	− 0.1059 (− 0.4070, 0.1953	ns	− 0.0118 (− 0.1615, 0.1380)	ns
MLP + P	3.25 (0.69)	3.28 (0.66)	3.33 (0.70)	0.0313 (− 0.2252, 0.2877)	ns	0.0750 (− 0.1498, 0.2998)	ns
*LDL/HDL ratio*							
Control	2.04 (0.38)	1.96 (0.42)	2.07 (0.47)	− 0.0786 (− 0.2358, 0.07862)	ns	0.0286 (− 0.09004, 0.1472)	ns
MLP + BioPB	2.05 (0.75)	1.92 (0.76)	2.04 (0.75)	− 0.1235 (− 0.4013, 0.1543)	ns	− 0.0059–0.1425 0.1307	ns
MLP + P	1.98 (0.56)	2.03 (0.56)	2.09 (0.62)	0.0500 (− 0.1503, 0.2503)	ns	0.1063 (− 0.0983, 0.3108)	ns
*Triglyceride/HDL ratio*							
Control	0.54 (0.23)	0.49 (0.22)	0.61 (0.30)	− 0.0500 (− 0.1735, 0.0735)	ns	0.0714 (− 0.0576, 0.2004)	ns
MLP + BioPB	0.55 (0.38)	0.58 (0.34)	0.58 (42)	0.0294 (− 0.0823, 0.1411)	ns	0.0294 (− 0.0777, 0.1366)	ns
MLP + P	0.58 (0.38)	0.57 (0.30)	0.54 (0.25)	− 0.0063 (− 0.1613, 0.1488)	ns	− 0.0313 (− 0.1765, 0.1140)	ns
*High-sensitivity CRP (mg/L)*							
Control	1.8 (1.73)	1.9 (2.04)	2.04 (2.27)	− 0.0679 (− 0.3868, 0.2511)	ns	− 0.1529 (− 0.7305, 0.4247)	0.0050
MLP + BioPB	1.56 (2.09)	0.91 (0.86)	1.45 (1.51)	− 0.1765 (− 0.4949, 0.1419)	ns	0.4418 (− 0.2901, 1.174)	ns
MLP + P	1.82 (1.52)	0.95 (0.74)	1.69 (1.20)	− 0.8669 (− 1.597, − 0.1367)	0.0094	− 0.1256 (− 1.160, 0.9089)	ns
*Glucose (fasting; mmol/L)*							
Control	5.26 (0.78)	5.29 (0.70)	5.34 (0.70)	0.0286 (− 0.1628, 0.2200)	ns	0.0714 (− 0.1075, 0.2504)	ns
MLP + BioPB	5.31 (0.55)	5.31 (0.49)	5.22 (0.51)	0.0059 (− 0.2450, 0.2568)	ns	− 0.0824 (− 0.3044, 0.1397)	ns
MLP + P	5.52 (0.77)	5.43 (0.59)	5.36 (0.76)	− 0.0938 (− 0.3134, 0.1259	ns	− 0.1625 (− 0.5116, 0.1866)	ns
*Insulin (fasting; mU/L)*							
Control	6.00 (3.04)	7.01 (3.98)	6.61 (3.37)	1.007 (− 0.3705, 2.385)	ns	0.6143 (− 0.6726, 1.901)	ns
MLP + BioPB	6.88 (4.30)	6.93 (4.28)	6.21 (4.11)	0.0529 (− 1.314, 1.420	ns	− 0.6706 (− 2.859, 1.518)	ns
MLP + P	7.87 (5.32)	6.48 (1.72)	8.00 (3.45)	− 1.394 (− 3.756, 0.9683)	ns	0.1313 (− 2.192, 2.455)	ns
*HOMA-IR score*							
Control	1.39 (0.65)	1.63 (0.85)	1.59 (0.88)	0.2429 (− 0.0460, 0.5317)	ns	0.2000 (− 0.1171, 0.5171)	ns
MLP + BioPB	1.65 (1.10)	1.67 (1.19)	1.47 (1.05)	0.0235 (− 0.3089, 0.3560)	ns	− 0.1824 (− 0.7365, 0.3718)	ns
MLP + P	1.98 (1.45)	1.56 (0.42)	1.96 (0.93)	− 0.4125 (− 1.051, 0.2263)	ns	− 0.0125 (− 0.6115, 0.5865)	ns

Trial details are described in Materials and Methods. Data represent the mean ± SD at baseline (Week 0), immediately after the intervention (Week 4) and 4 weeks postintervention follow-up (Week 8), the mean differences (Δ) with the 95% CI from baseline and *p* values at Week 4 and Week 8. Differences within groups across times were analysed using the Friedman test, a non-parametric repeated measures one-way ANOVA followed by Dunn’s post hoc test using GraphPad Prism 10.4.2. Differences were considered significant if *p* < 0.05

#### Glucose and insulin LDL

Fasting glucose and insulin were similar between baseline, Week 4, and Week 8 in all groups (Table 2). Both MLP + P and MLP + BioPB groups showed non-significant improvements in HOMA-IR. Fasting insulin and HOMA-IR scores were also similar between groups at all observation time points. No significant differences were found between groups at any time point.

#### Inflammation

High-sensitivity CRP showed a significant reduction (*p* = *0.0094*) in the MLP + P group and a non-significant reduction in the BioPB group at Week 4 compared to baseline. No statistically significant differences were found for any of the other groups at Week 8 (Table 2). There was no significant difference in hs-CRP between groups at any observation time.

#### Liver and kidney function

Changes in liver and kidney function biomarkers are presented in Table S2. Compared to baseline, significant reductions in ALP were observed at Week 4 in both MLP + BioPB (*p* < 0.0001) and MLP + P groups (*p* < 0.01). GGT levels were also reduced in MLP + BioPB and MLP + P at Week 4 compared to baseline; however, only the reduction in the MLP + BioPB group was statistically significant (*p* < 0.01). Following the intervention, significant reductions in creatinine levels were observed at Week 8 in MLP + BioPB (*p* < 0.05) and MLP + P (*p* < 0.05). Similarly, decreases in the levels of AST were also observed at Week 8 (all groups) and in the Control and MLP + P groups at Week 4.

#### Blood pressure and heart rate

There was no significant difference in blood pressure and heart rate between and within groups at any of the time points (Supplementary Table S3).

### Gastrointestinal symptoms and adverse events

#### Stool type

Stool type was assessed by participants using the Bristol Stool Chart. The chart categorises stools into seven different types based on their appearance, consistency, and shape, which can provide valuable information about a person’s digestive health [62].

At baseline, most participants in the control and MLP + P groups indicated having a normal stool (Type 3 or 4; Supplementary Fig. S2). In contrast, for an unknown reason, there was greater variability in stool type at baseline for the MLP + BioPB group. In the MLP + P group, relatively fewer participants reported normal stools and more reported stools that were either softer (type 7: 1 of 16 compared to 0 of 17 at Week 0) or harder (Types 1, 2: 6 of 16 compared to 3 of 17 at Week 0) at Week 4. There was no major difference in reported stool type in the Control group at Week 4 compared to baseline. In the MLP + BioPB group, slightly more participants reported Type 2 or Type 5 stool types at baseline. In comparison to baseline, however, more participants reported a ‘normal’ stool type (Type 3 or 4) at Week 4. However, the differences were small.

#### Gastrointestinal tolerability

Tolerability was assessed using the Gastrointestinal Symptoms and Stool Output questionnaire. There was no significant difference in GI Symptom Scores between baseline and Week 4 in any group (data not shown). Further, there was no significant difference in the GI Symptoms Score at Week 0, 4, or 8 between any of the groups.

### Health-related quality of life

There was a significant increase in the RAND score in the MLP + BioPB group at weeks 4 and 8 compared to the baseline (Supplementary Fig. S3). The Control group also showed a significant increase in RAND score compared to the baseline at Week 8. No differences in RAND scores were observed for MLP + P.

#### Reported side effects

The side effects recorded over all groups included nausea, stomach pain, abdominal discomfort/cramps, bloating, diarrhoea, flatulence, and constipation. The control and MLP + BioPB groups showed no significant difference in total side effect scores between baseline, intervention, and follow-up. However, significant improvements in the numbers of subjects reporting side effects at Week 5 and Week 8, compared to baseline, were observed in the MLP + BioPB and MLP + P groups (Supplementary Tables S4–S10). There was no significant difference in the overall side effect score between groups at any of the time points.

## Discussion

The results of the present study demonstrate that the MLP combining TRE, dietary fibre, probiotics and chromium, over a short period, was highly effective in lowering body weight and improving body composition in healthy overweight individuals. Some improvements in blood cholesterol and LDL profiles and markers of liver and kidney functions were also observed in subjects receiving MLP + BioPB. None of the interventions had any effect on glucose and insulin homeostasis.

Subjects receiving MLP + P or MLP + BioPB lost on average 4.8 kg (5.9%) or 5.1 kg (6.1%) body weight, respectively, within a short period of four weeks. Furthermore, the weight loss was sustained for a further 4 weeks after cessation of the intervention. A reduction of 5% in body weight is considered clinically significant, and this has been shown to reduce the risk of metabolic disorders by improving blood lipids and glucose homeostasis [67–69]. Considering that each kilogram of weight loss in an overweight person leads to a 16% reduction in the risk of type-2 diabetes [70], our findings confirm the efficacy of a multifaceted approach in reducing the risk and incidence of metabolic disease in at-risk populations. Conventional weight loss programs have shown variable effectiveness in reducing body weight, ranging from 1.1 kg for exercise (3–12 months [71]), 4.6 kg for diet (12 months [72]), and 2.6 to 8.8 kg for pharmacotherapies [73]. The average weight reduction of 5 kg observed in our study over a short period suggests a higher efficacy of the present intervention. Whether the weight reduction effect was due to synergistic effects between dietary changes, fibre, TRE, probiotics, or chromium in one or other combinations cannot be ascertained within this multicomponent intervention. Nonetheless, the dietary advice, dietary fibre and TRE are likely to be the major contributors; the evidence for the efficacy of probiotics and chromium has been relatively weak and highly variable [48, 74].

Many studies have reported that increasing dietary fibre intake leads to increased satiety, reduced appetite, reduced energy consumption and weight loss [75]. A negative correlation between total dietary fibre and body weight has also been reported [76, 77]. The effect of dietary fibre has also been found to vary depending on the source and type of dietary fibre [78–80], and differences in individual responsiveness to dietary fibre intake have also been reported, though more research is needed in this area [79]. The optimum duration for dietary fibre intervention remains unknown. A meta-analysis by Huwiler et al. (2022) concluded that increased dietary fibre intake for at least 12 weeks is required to realise any significant effect [81]. Thus, in the present study, a longer intervention may have produced a better result. Nevertheless, meta-analysis and systematic reviews by others [32, 82] found no convincing evidence for the effectiveness of dietary fibre alone in lowering body weight and an inconsistent effect of dietary fibre intake on weight loss was also reported by Chen et al., 2018 [33].

The MLP also led to significant reductions in BMI, waist-to-hip ratio, waist-to-height ratio, waist circumference, % body fat, and visceral fat in the study participants. This proves the effectiveness of these interventions in improving adiposity, especially abdominal obesity, a major risk factor for metabolic syndrome. These findings are consistent with the results of previous studies that have examined the effect of MLP, TRE or calorie-restricted diets, dietary fibre, chromium, and some probiotics leading to improvements in body composition [21, 22, 83, 84]. However, the magnitude of these changes was much greater in our study, possibly due to the cumulative effects of the specific components of the intervention as noted. Furthermore, TRE is often associated with the loss of lean tissue mass [23, 24]. Although subjects in both the intervention groups exhibited significant decreases in skeletal muscle mass, soft lean mass, fat fat-free mass, these were lower by a factor of 6 to 10 compared to reductions in the visceral and total body fat. This suggests that most of the weight loss was attributable to fat loss. Nonetheless, it also highlights that future MLP interventions may benefit from additional protein supplementation to counter lean tissue mass loss.

The significant improvements in the anthropometric measurements and body composition in the MLP + P and MLP + BioPB groups were associated with reduced consumption of sugary foods and increased consumption of non-starchy vegetables, meat and eggs. Lowering sugar intake and increasing the consumption of non-starchy vegetables is known to improve glucose and insulin homeostasis [85], reduce calorie intake, promote satiety and thus reduce body weight in obese subjects [86–88]. Reducing calorie intake to achieve a negative calorie balance is considered critical for weight loss [89], but ensuring healthy outcomes is not so simple. The increased consumption of non-starch vegetables would also have mediated a beneficial effect by increasing the consumption of dietary fibre and modulating the composition of the gut microbiota [90, 91].

Some but not all lifestyle modification programs, TRE, dietary fibre, probiotics, and chromium interventions have been reported to be effective in improving glucose regulation [19, 23, 78, 92]. The present intervention, although highly effective in weight reduction, had limited (non-significant) effects on fasting blood glucose and insulin levels and HOMA-IR. This could be due to two main reasons. First, the inclusion of participants who were overweight but healthy with no comorbidities (fasting glucose and insulin levels of the study subjects at the baseline were in the normal range). Second, the four-week duration of the intervention was possibly too short to observe further significant changes. Studies that have shown improvements in glycaemic responses require much longer interventions to be manifested [93, 94]. Further long-term studies in overweight or obese subjects are needed to clarify this.

Another interesting feature of this study was the observed differing effects of psyllium and BioPB fibres included in the MLP on cardiovascular disease risk factors. MLP + BioPB, but not MLP + P, was highly effective in modulating blood lipids. Compared to the baseline, the subjects in the MLP + BioPB group had significantly lower levels of total cholesterol, non-HDL cholesterol and LDL post-intervention at Weeks 4 and 8. Although the observed effects for MLP + BioPB were small, these effects are clinically relevant. The differential effect between the two interventions appears to be solely due to differences in the source and type of fibres. This is likely due to each dietary fibre having distinct physicochemical properties [75]. In contrast, the lack of any effect resulting from psyllium supplementation is at odds with the results of several other studies. For example, a meta-analysis by Brown et al. [95] found that each gram of fibre from psyllium was associated with a reduction of 0.028 mmol/L cholesterol and 0.029 mmol/L of LDL concentration. Soluble dietary fibre, a major component of psyllium fibre, has also been suggested to mediate a hypocholesterolemic effect by reducing cholesterol absorption and bile salt reabsorption [92, 96]. Whether the lack of effect in our study was due to a lower dose of psyllium (8.4 g/day) or some other reason is not known.

On the other hand, psyllium was effective in reducing hs-CRP concentrations since these were significantly reduced in the MLP + P group but not in the MLP + BioPB group. CRP is a well-recognised indicator of systemic inflammation, and chronic low-grade inflammation is considered a key contributor to the development of metabolic syndrome [97, 98]. The accumulation of visceral fat observed in central obesity is associated with increased CRP levels mediated by the release of proinflammatory cytokines from the fat tissue [99]. The significant decrease in CRP levels observed in the MLP + P group is indicative of a reduction in systemic inflammation, probably driven by visceral and total fat loss. This again highlights the disparate and selective health effects of different dietary fibres and suggests that an MLP incorporating psyllium supplementation can attenuate inflammation and improve metabolic health. These findings are in line with previous studies that have reported the effectiveness of lifestyle modification programs or certain types of dietary fibres in reducing body weight and CRP concentrations [100, 101].

This study also revealed significant improvements in liver and kidney functions in the MLP + P and MLP + BioPB groups, evidenced by reductions in the ALP, GGT, and creatinine levels. These observations are clinically relevant given the association between elevated liver enzymes and non-alcoholic fatty liver disease and are consistent with the idea that dietary and lifestyle interventions can be used to improve liver health by reducing visceral fat and liver inflammation [102, 103]. The decrease in creatinine levels suggests improvements in kidney function and/or a reduction in muscle catabolism. These findings are consistent with previous studies reporting improvements in liver [104] and kidney functions through interventions aimed at metabolic health improvement [83].

Age is unlikely to have significantly influenced the observed changes in weight and body composition in response to MLP + BioPB and MLP + P supplementation. The average age of participants enrolled in the study was 48.9 years (range 28–62), placing the cohort predominantly within the age range (20–60 years) during which metabolism remains stable [105]. Given that metabolic rate begins to decline (by 0.7% per annum) after the age of 60, older subjects likely exhibited a slightly diminished response to the intervention. However, given the small number of subjects between 60 and 62 years in our study, any such effect is likely to be minimal overall. This conclusion is also supported by the findings of Bouchard et al. [106] who reported that lifestyle interventions in older individuals conferred equal or greater improvements in body weight, blood pressure and glycaemic control than those observed in younger individuals.

All interventions included in the study were generally well tolerated. Apart from a few reports of harder stools in a small number of participants (3 of 17 at Week 0 versus 6 of 16 at Week 4) in the MLP + P group, improvements in indicators of gut health and Health Quality scores were observed in both intervention groups.

The limitations of this study included a relatively short intervention period of 4 weeks and a small sample size. Other significant limitations of the study were the use of self-reported dietary and physical activity data, leading to personal biases and inaccurate and incomplete data. The requirement for subjects to sprinkle the supplemental dietary fibre on food twice a day, and to take the probiotics daily, may also have affected compliance.

In summary, MLP combining TRE, dietary fibre (psyllium or BioPB), probiotics and chromium supplementation, is a highly effective strategy in modifying risk factors associated with metabolic syndrome (body weight, BMI, waist circumference, body fat mass and visceral fat) in overweight subjects. The MLP, regardless of fibre type, also led to significant improvements in liver and kidney functions. Notwithstanding, MLPs incorporating different fibres exhibited varying effects on blood lipids and inflammation. Specifically, MLP + BioPB was effective in reducing cholesterol and LDL, while MLP + P was effective in reducing inflammation. Given the characteristics of our study population (healthy, overweight participants with no metabolic abnormalities), these findings are likely to apply to a broader population of obese individuals and those with metabolic abnormalities. Further well-designed, long-term studies, especially in obese participants, are needed to determine the effectiveness of these interventions for sustained weight loss and improvements in biomarkers of metabolic homeostasis in diverse population groups.

## Supplementary Information

Below is the link to the electronic supplementary material.

Supplementary file1 (DOCX 724 KB)

## Data Availability

Data described in the manuscript will be made available on request (subjects will remain de-identified).

## Abbreviations

BioPB: A novel dietary fibre consisting of equal proportions of carrageenan, konjac, and cellulose
Hs-CRP: High-sensitivity C-reactive protein
MLP: Multifaceted lifestyle program
MLP+BioPB: Multifaceted lifestyle program with BioPB novel dietary fibre
MLP+P: Multifaceted lifestyle program with psyllium
BMI: Body mass index
PBF: Percentage body fat
SMM: Skeletal muscle mass
