# Supplementary material for: A multifaceted lifestyle program for weight loss in overweight adults: evidence from a randomised clinical trial
Source: Eur J Nutr. 2026 Jan 12;65(1):22. doi: 10.1007/s00394-025-03873-w (PMC12795926; doi:10.1007/s00394-025-03873-w)
Supplement: Supplementary file 1 — Supplementary file1 (DOCX 724 KB) [file 394_2025_3873_MOESM1_ESM.docx]

**Supplementary Information**

**A multifaceted lifestyle program for weight loss in overweight adults: evidence from a randomized clinical trial​**

**European Journal of Nutrition**

​​Harsharn Gill ^1^*, Chintha Lankatillake ^1,2^, Elena Zafiris^1^ and Christopher Pillidge^1^,​

​​^1^ School of Science, RMIT University, Melbourne, Victoria, Australia

​^2^ School of Health and Biomedical Sciences, RMIT University, Bundoora, VIC, Australia​

**​​**

**​***Correspondence: Harsharn Gill, harsharn.gill@rmit.edu.au

# Supplementary Questionnaires

**Questionnaire S1.** RAND 36 Item Short-form Health Survey

Welcome to the RAND 36 Item SF Health Survey.

The purpose of this survey is to assess your perceived health status and health-related quality of life.

1. In general, would you say your health is: 1 Excellent
   1. Very good


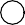

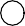

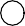

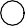

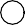


- 1. Good
  2. Fair
  3. Poor

1. Compared to one year ago, how would your rate your 1 Much better now than one year ago health in general now? 2 Somewhat better now than one year ago
2. About the same


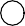

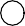

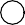

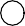

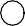


1. Somewhat worse now than one year ago
2. Much worse now than one year ago

**The following items are about activities you might do during a typical day. Does your health**

**now limit you in these activities? If so, how much? (Choose one answer for each question.)**

1. Vigorous activities, such as running, lifting heavy 1 Yes, limited a lot objects, participating in strenuous sports 2 Yes, limited a little

3 No, not limited at all


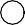

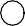

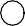


1. Moderate activities, such as moving a table, 1 Yes, limited a lot pushing a vacuum cleaner, bowling, or playing golf 2 Yes, limited a little

3 No, not limited at all


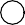

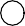

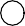


1. Lifting or carrying groceries 1 Yes, limited a lot
   1. Yes, limited a little


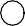

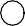

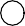


- 1. No, not limited at all

1. Climbing several flights of stairs 1 Yes, limited a lot
   1. Yes, limited a little


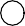

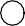

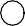


- 1. No, not limited at all

1. Climbing one flight of stairs 1 Yes, limited a lot
   1. Yes, limited a little


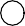

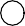

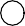


- 1. No, not limited at all

1. Bending, kneeling, or stooping 1 Yes, limited a lot
   1. Yes, limited a little


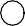

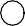

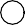


- 1. No, not limited at all

1. Walking more than a mile (about 1.6 km) 1 Yes, limited a lot
   1. Yes, limited a little


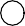

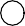

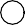


- 1. No, not limited at all

1. Walking several blocks 1 Yes, limited a lot
   1. Yes, limited a little


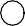

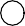

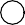


- 1. No, not limited at all

1. Walking one block 1 Yes, limited a lot
   1. Yes, limited a little


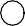

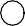

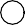


- 1. No, not limited at all

1. Bathing or dressing yourself 1 Yes, limited a lot


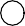

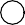

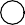


- 1. Yes, limited a little
  2. No, not limited at all


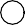

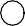


**During the past 4 weeks, have you had any of the following problems with your work or other regular daily activities as a result of your physical health? (Choose one answer for each**

**question.)**

1. Cut down the amount of time you spent on work or 1 Yes other activities 2 No
2. Accomplished less than you would like 1 Yes 2 No
3. Were limited in the kind of work or other 1 Yes

activities 2 No


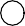

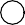

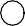

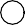


1. Had difficulty performing the work or other 1 Yes

activities (for example, it took extra effort) 2 No


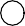

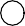

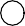

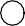


**During the past 4 weeks, have you had any of the following problems with your work or other regular daily activities as a result of any emotional problems (such as feeling depressed or**

**anxious)? (Choose one answer for each question.)**

1. Cut down the amount of time you spent on work or 1 Yes other activities 2 No
2. Accomplished less than you would like 1 Yes 2 No
3. Didn't do work or other activities as carefully as 1 Yes

usual 2 No


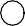

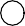

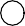

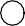


1. During the past 4 weeks, to what extent has your 1 Not at all physical health or emotional problems interfered with 2 Slightly your normal social activities with family, friends, 3 Moderately

neighbors, or groups? (Choose one answer.) 4 Quite a bit 5 Extremely


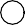

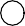

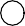

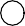

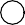


1. How much bodily pain have you had during the past 1 None

4 weeks? (Choose one answer.) 2 Very mild


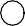

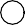

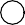

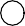

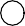

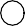


3 Mild

4 Moderate

5 Severe

6 Very severe

1. During the past 4 weeks, how much did pain 1 Not at all interfere with your normal work (including both work 2 A little bit outside the home and housework)? (Choose one answer.) 3 Moderately

4 Quite a bit


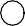

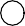

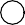

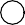

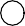


5 Extremely

**These questions are about how you feel and how things have been with you during the past 4 weeks. For each question, please give the one answer that comes closest to the way you have been feeling.**

**How much of the time during the past 4 weeks . . .(Choose one answer for each question.)**

1. Did you feel full of pep? 1 All of the time


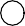

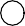

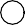

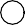

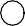

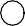


- 1. Most of the time
  2. A good bit of the time
  3. Some of the time
  4. A little of the time
  5. None of the time

1. Have you been a very nervous person? 1 All of the time
   1. Most of the time


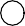

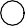

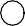

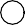

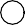

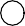


- 1. A good bit of the time
  2. Some of the time
  3. A little of the time
  4. None of the time

1. Have you felt so down in the dumps that nothing 1 All of the time could cheer you up? 2 Most of the time
2. A good bit of the time


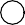

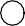

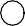

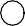

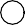

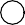


1. Some of the time
2. A little of the time
3. None of the time
4. Have you felt calm and peaceful? 1 All of the time
   1. Most of the time


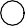

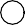

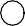

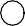

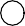

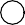


- 1. A good bit of the time
  2. Some of the time
  3. A little of the time
  4. None of the time

1. Did you have a lot of energy? 1 All of the time
   1. Most of the time


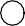

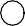

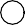

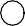

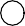

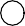


- 1. A good bit of the time
  2. Some of the time
  3. A little of the time
  4. None of the time

1. Have you felt downhearted and blue? 1 All of the time
   1. Most of the time

- 1. A good bit of the time
  2. Some of the time
  3. A little of the time
  4. None of the time

1. Did you feel worn out? 1 All of the time
   1. Most of the time

- 1. A good bit of the time
  2. Some of the time
  3. A little of the time
  4. None of the time

1. Have you been a happy person? 1 All of the time

- 1. Most of the time
  2. A good bit of the time
  3. Some of the time
  4. A little of the time
  5. None of the time

1. Did you feel tired? 1 All of the time
   1. Most of the time

- 1. A good bit of the time
  2. Some of the time
  3. A little of the time
  4. None of the time

1. During the past 4 weeks, how much of the time has 1 All of the time your physical health or emotional problems interfered 2 Most of the time with your social activities (like visiting with 3 Some of the time

friends, relatives, etc.)? 4 A little of the time

5 None of the time

(Choose one number)

**How TRUE or FALSE is each of the following statements for you.**

**(Choose one number for each question.)**

1. I seem to get sick a little easier than other 1 Definitely true

people 2 Mostly true

1. Don't know
2. Mostly false
3. Definitely false
4. I am as healthy as anybody I know 1 Definitely true
   1. Mostly true

- 1. Don't know
  2. Mostly false
  3. Definitely false

1. I expect my health to get worse 1 Definitely true
   1. Mostly true

- 1. Don't know
  2. Mostly false
  3. Definitely false

1. My health is excellent 1 Definitely true
   1. Mostly true

- 1. Don't know
  2. Mostly false
  3. Definitely false

RAND® is a registered trademark. Copyright © 1994-2010 RAND Corporation. RAND Health is a research division of the RAND Corporation. Used with permission

<http://www.rand.org/health/surveys_tools/mos/mos_core_36item.html>

**Questionnaire S2.** Gastrointestinal Symptoms and Stool Output

Welcome to the Gastrointestinal Symptoms and Stool Output Questionnaire. This questionnaire is designed assess your gut health and function.

Abdominal pains. No or transient pain

Rate according to intensity, frequency, duration, Occasional aches and pains interfering with some request for relief, and impact on social performance. social activities

Prolonged and troublesome aches and pains causing requests for relief and interfering with many

social activities

Severe or crippling pains with an impact on all social activities

Heartburn. No or transient heartburn

Rate according to intensity, frequency, duration, and Occasional discomfort of short duration request for relief. Frequent episodes of prolonged discomfort;

requests for relief

Continuous discomfort with only transient relief by antacids

Reflux (sudden regurgitation of acid gastric content). No or transient regurgitation

Occasional troublesome regurgitation

Rate according to intensity, frequency, and request Regurgitation once or twice a day; requests for for relief. relief

Regurgitation several times a day; only transient and insignificant relief by antacids

Sucking sensations in the epigastrium. No or transient sucking sensation Representing a sucking sensation in the epigastrium Occasional discomfort of short duration; no with relief by food or antacids. If food or antacids requests for food or antacids between meals are not available, the sucking sensations progress to Frequent episodes of prolonged discomfort; aches and pains. Rate according to intensity, requests for food and antacids between meals

frequency, duration, and request for relief. Continuous discomfort; frequent requests for food or antacids between meals

The epigastrium is the upper central region of the abdomen, located between the lower edges of the rib cage and the belly button.

Nausea and vomiting. No nausea

Occasional episodes of short duration

Rate according to intensity, frequency, and duration. Frequent and prolonged nausea; no vomiting

Continuous nausea; frequent vomiting

Abdominal rumbling/gurgling (borborygmus). No or transient borborygmus

Occasional troublesome borborygmus of short

Rate according to intensity, frequency, duration, and duration

impact on social performance. Frequent and prolonged episodes which can be mastered by moving without impairing social performance

Continuous borborygmus severely interfering with social performance

Bloating (bloating with abdominal gas). No or transient distension

Rate according to intensity, frequency, duration, and Occasional discomfort of short duration

impact on social performance. Frequent and prolonged episodes which can be mastered by adjusting the clothing

Continuous discomfort seriously interfering with social performance

Belching (eructation). No or transient eructation Occasional troublesome eructation

Rate according to intensity, frequency, and impact on Frequent episodes interfering with some social social performance. activities

Frequent episodes seriously interfering with social performance

Flatulence (passing wind). No increased flatulence

Occasional discomfort of short duration

Rate according to intensity, frequency, duration, and Frequent and prolonged episodes interfering with impact on social performance. some social activities

Frequent episodes seriously interfering with social performance

Stool frequency. Once a day

Every third day If you have a stool frequency of more than once a day, Every fifth day

this will be assessed in the next question. Every seventh day or less frequently

Increased passage of stools. Once a day

Rate according to frequency. Three times a day Five times a day

Seven times a day or more frequently

Loose stools. Normal consistency

Rate according to consistency independent of frequency Somewhat loose and feelings of incomplete evacuation. Runny

Watery

Hard Stools. Normal consistency

Rate according to consistency independent of frequency Somewhat hard and feelings of incomplete evacuation. Hard

Hard and fragmented, sometimes in combination with diarrhoea

Urgent need for defecation (feelings of incomplete Normal control

control and inability to control defecation). Occasional feelings of urgent need for defecation Frequent feelings of urgent need for defecation

Rate according to intensity, frequency, and impact on with sudden need for a toilet interfering with social performance. social performance

Inability to control defecation

Feeling of incomplete evacuation (defecation with Feeling of complete evacuation without straining straining and a feeling of incomplete evacuation of Defecation somewhat difficult; occasional feelings stools). of incomplete evacuation

Rate according to intensity and frequency. Defecation difficult; often feelings of incomplete evacuation

Defecation extremely difficult; regular feelings of incomplete evacuation

The Bristol Stool Form Scale (BSFS) is a tool used to classify the consistency of human faeces into seven different types based on their shape and texture. It is widely used to evaluate digestive health.

Type of stool (stool consistency).

Please select stool consistency based on the Bristol Stool Form Scale.

**Questionnaire S3.** Potential Side-Effects

Please note: if you are completing this questionnaire for Week 0, this is your Pre-Existing Symptoms Questionnaire. Welcome to the Potential Side Effects Questionnaire!

The purpose of this questionnaire is to monitor whether you experience any side effects from your supplementation.

Please rate the frequency of the following symptoms, over the past week, on a scale of 0 - 5 where:

0 = none

1 = minimal (1-2 times per week)

2 = slight (3-4 times per week)

3 = occasional (5-6 times per week)

4 = frequent (7-8 times per week)

5 = severe (9 or more times per week)

none minimal (1-2

times per week)

slight (3-4

times per week)

occasional

(5-6 times per week)

frequent (7-8

times per week)

severe (9 or

more times per week)

1. Nausea
2. Stomach pain
3. Abdominal discomfort/cramps
4. Bloating
5. Diarrhoea
6. Flatulence (passing gas)
7. Constipation
8. Any other unusual effects? Yes No
9. Please specify other unusual effect(s):
10. Please rate any other unusual or adverse effects that none

you may have experienced. minimal (1-2 times per week) slight (3-4 times per week) occasional (5-6 times per week) frequent (7-8 times per week) severe (9 or more times per week)

# Supplementary Methods

**Physical Activity Calculations**

Physical activity data were standardized to minutes per day, and where activity durations were missing, standardized assumptions were applied. Physical activity times (minutes) were used to calculate the mean daily exercise duration for each timepoint, for each participant. Group means were derived by averaging participants’ data within each group

**Activity specific estimates:**

When physical activity was reported without a duration, the following estimates were applied:

- Running: 6 minutes per km for running (1)
- Walking: 11.34 minutes per km for walking (2)
- Cycling: 2.1 minutes per km for cycling (3)
- Steps: 51.9 seconds (0.865min) per 100 steps for step counts (4)

A total 20 minutes per session were assigned for physical activities that were not recorded with a time or distance component, e.g. ‘yoga’, ‘gym’, or’ swimming’.

Incidental activities not related to purposeful exercise (e.g., "craft," "busy day") were excluded.

To estimate the time required to complete 100 steps, we utilized the following logic based on average walking speeds and stride lengths:

- Walking Speed Assumption: The average walking speed for adults at a moderate pace was assumed to be 1.47 m/s (mean speed for medium-paced walking).
- Stride Length Assumption: Based on existing literature, the average stride length for an adult is approximately 0.762 meters (76.2 cm).
- Steps per km Calculation: Given the average stride length, the number of steps per kilometer can be calculated as: Steps per km =1000 m / (0.762m/step) ≈ 1312 steps/km
- Time to Walk 1 km: At a walking speed of 1.47m/s, time per step = 11.34 min / 1312 steps ≈ 0.00865 min/ step ( or 0.519 sec/step)

# Supplementary Figures

**Figure S1.** Physical Activity Data (in minutes) Across Intervention Arms

This figure displays the average daily physical activity duration, in minutes, for each arm of the intervention at each timepoint (Week 0, Week 4, Week 8). Physical activity data was standardized into minutes per day, with durations calculated based on reported activity and established assumptions for missing data as per Supplementary Methods. Group means were derived by averaging individual partipants data within each group at each time point. This approach ensures consistent comparisons of physical activity pattersna dn changes over time within and between intervention groups.

**Figure S2.** Stool type between weeks 0 – 8.

**Figure S3.** Comparison of RAND scores at baseline (Week 0), end of the intervention period (Week 4), and at the end of the follow-up period (Week 8). Data represents mean + SD. Differences between weeks were determined by Friedman’s test followed by Dunn’s post hoc test. Differences were considered significant if P < 0.05.

# Supplementary Tables

**Table S1.** Anthropometric and body composition measurements at Weeks 0 (preintervention), 4 (post-intervention) and 8 (4 weeks after the intervention had ceased), and changes from baseline (Δ) at Weeks 4 and 8. The trial consisted of three groups: a control group that received a placebo (rice powder; n=16), the MLP+BioPB group, which received the MLP with BioPB (n=18), and the MLP+P group which received the MLP with psyllium (n=16). Data represents the mean ± SD at baseline (Week 0), after the intervention (Week 4) and 4 weeks postintervention (Week 8), and the mean differences (Δ) with the 95% CI from baseline at Week 4 and Week 8 and per cent difference. The adjusted *p* values for comparisons at Week 4 and Week 8 relative to baseline are also included to demonstrate the clinical significance of the observed changes and the impact of the interventions. Differences between groups were analysed using Friedman ANOVA followed by Dunn’s post hoc test using GraphPad Prism 10.0.2. Differences were considered significant if p < 0.05.

|  | **Mean ± (SD)** | | | **Mean Difference from Baseline (95% CI)** | | | | | |
| --- | --- | --- | --- | --- | --- | --- | --- | --- | --- |
|  | **Week 0** | **Week 4** | **Week 8** | **Week 4** | | | **Week 8** | | |
|  |  |  |  | **Mean Difference** | ***p* value** | **Percent Difference** | **Mean Difference** | ***p* value** | **Percent Difference** |
| **Weight (kg)** | | | | | | | | | |
| **Control** | 82.18 (12.71) | 81.84 (12.72) | 82.14 (13.31) | -0.3 (-1.1,to 0.4) | ns | -0.4 (-1.4, 0.6) | 0.0 (-1.2, 1.1) | ns | -0.1 (-1.6,1.4%) |
| **MLP+BioPB** | 81.57 (10.80) | 76.66 (9.889) | 76.05 (9.959) | -5.1 (-6,0, -4.1) | 0.0002 | -6.1(-7.2, -5.1) | -5.4 (-6.7, 4.1) | <0.0001 | -6.5 (-7.9, -5.1) |
| **MLP+P** | 80.33 (8.902) | 75.51 (7.503) | 75.16 (7.954) | -4.8 (-5.9,to -3.6) | 0.0002 | -5.9 (-7.2 to -4.6) | -5.3 (-7.0 to -3.5) | <0.0001 | -6.5 (-8.5, -4.5) |
| **Body Mass Index (kg/m^2)** | | | | | | | | | |
| **Control** | 28.51 (1.89) | 28.39 (1.775) | 28.48 (1.859) | -0.1 (-0.4, 0.2) | ns | -0.4 (-1.3, 0.6) | 0.0 (-0.5, 0.4) | ns | 0.0 (-1.5 1.4) |
| **MLP+BioPB** | 28.26 (1.835) | 26.58 (1.738) | 26.42 (1.771) | -1.7 (-2.0, -1.4) | 0.0002 | -6.1 (-7.1, -5.1) | -1.8 (-2.2, 1.4) | <0.0001 | -6.4 (-7.8, -5.1) |
| **MLP+P** | 28.12 (1.848) | 26.51 (1.594) | 26.46 ±1.934 | -1.7(-2.1, -1.3) | 0.0003 | -5.9 (-7.2, -5.1) | -1.8 (-2.4, -1.4) | <0.0001 | -6.2 (-8.4,-4.5) |
| **Waist to Hip ratio (cm)** | | | | | | | | | |
| **Control** | 0.956 (0.066) | 0.958 (0.065) | 0.955 (0.061) | 0.00(-0.01, 0.01) | ns | 0.15 (-0.77, 1.08) | 0.00(-0.01, 0.01) | ns | -0.07 (-1.26, 1.12) |
| **MLP+BioPB** | 0.973 (0.059) | 0.943 (0.056) | 0.940 (0.064) | -0.03 (-0.04, -0.02) | 0.0004 | -3.06 (-3.99, -2.12) | -0.03(-0.05, -0.02) | 0.0001 | -3.57(-4.82, -2.31) |
| **MLP+P** | 0.965 (0.054) | 0.950 (0.050) | 0.943 (0.054) | -0.02(-0.02, -0.01) | ns | -1.52 (-2.50, -0.54) | -0.02(-0.04, -0.01) | 0.0029 | -2.49(-3.85, -1.13) |
| **Waist to Height ratio (cm)** | | | | | | | | | |
| **Control** | 0.58 (0.042) | 0.58 (0.040) | 0.58 (0.038) | -0.02 (-0.96, 0.92) | ns | 0.00 (-0.01, 0.01) | 0.15 (-1.39, 1.68) | ns | -0.02 (-0.96, 0.92) |
| **MLP+BioPB** | 0.59 (0.035) | 0.56 (0.035) | 0.055 (0.041) | -0.03 (-0.04,-0.03) | 0.0002 | -5.66 (-6.8, -4.52) | -0.04 (-0.04, -0.03) | <0.0001 | -6.18 (-7.62, -4.74) |
| **MLP+P** | 0.59 (0.042) | 0.57 (0.037) | 0.56 (0.041) | -0.03 (-0.03,-0.02) | 0.0011 | -4.20 (-5.37, -3.03) | -0.03 (-0.04, -0.02) | 0.0001 | -5.32 (-7.37,-3.27) |
| **Neck Circumference (cm)** | | | | | | | | | |
| **Control** | 37.67 (2.487) | 37.65 (2.424) | 37.74 (2.486) | -0.02 (-0.25, 0.21) | ns | -0.03 (-0.67, 0.61) | 0.08 (-0.3, 0.4) | ns | 0.22 (-0.72, 1.15) |
| **MLP+BioPB** | 37.61 (1.962) | 36.67 (1.904) | 36.51 (2.042) | -0.95 (-1.2, -0.7) | 0.0029 | -2.51 (-3.22, -1.79) | -1.15 (-1.5, -0.8) | <0.0001 | -3.1 (-4.01, -2.11) |
| **MLP+P** | 37.39 (2.272) | 36.54 (1.985) | 36.40 (2.124) | -0.83 (-1.2, -3.63) | 0.0011 | -2.2 (-3.03, -1.33) | -1 (-1.4, -0.6) | 0.0004 | -2.64 (-3.6, -1.69) |
| **Chest Circumference (cm)** | | | | | | | | | |
| **Control** | 102.4 (6.625) | 102.2 (6.761) | 102.7 (6.983) | -0.26 (-0.70, 0.17) | ns | -0.26 (-0.69, 0.17) | 0.24 (-0.6, 1.1) | ns | 0.22 (-0.58, 1.03) |
| **MLP+BioPB** | 102.4 (5.873) | 99.86 (5.905) | 99.64 (6.121) | -2.57 (-3.1, -2.1) | <0.0001 | -2.51 (-3.00, -2.03) | -2.82 (-3.5, -2.2) | <0.0001 | -2.76 (-3.40, -2.12) |
| **MLP+P** | 100.6 (5.015) | 98.49 (4.821) | 98.14 (4.883) | -2.26 (-3.1,-1.4) | 0.0004 | -2.2 (-3.01, -1.42) | -2.64 (-3.7, -1.6) | 0.0004 | -2.59 (-3.61, -1.57) |

**T**

|  | **Mean ± (SD)** | | | **Mean Difference from Baseline (95% CI)** | | | | | |
| --- | --- | --- | --- | --- | --- | --- | --- | --- | --- |
|  | **Week 0** | **Week 4** | **Week 8** | **Week 4** | | | **Week 8** | | |
|  |  |  |  | **Mean Difference** | ***p* value** | **Percent Difference** | **Mean Difference** | ***p* value** | **Percent Difference** |
| **Waist Circumference (cm)** | | | | | | | | | |
| **Control** | 98.74 (7.890) | 98.73 (8.113) | 98.89 (8.460) | -0.02 (-0.95, 0.91) | ns | -0.02 (-0.96, 0.92) | 0.14 (-1.4, 1.6) | ns | 0.15 (-1.39, 1.68) |
| **MLP + BioPB** | 100.0 (8.402) | 94.47 (7.629) | 93.92 (8.513) | -5.72 (-6.9, -4.5) | 0.0002 | -5.66 (-6.80, -4.52) | -6.19 (-7.7, -4.7) | <0.0001 | -6.18 (-7.62, -4.74) |
| **MLP + P** | 99.26 (8.091) | 94.89 (6.740) | 94.04 (7.371) | -4.24 (-5.5, -3.01) | 0.0006 | -4.2 (-5.37, -3.03) | -5.36 (-7.5, -3.3) | <0.0001 | -5.32 (-7.37, -3.27) |
| **Hip Circumference (cm)** | | | | | | | | | |
| **Control** | 103.3 (4.430) | 103.2 (4.424) | 103.4 (4.586) | -0.07 (-0.54, 0.41) | ns | -0.06 (-0.52, 0.39) | 0.1 (-0.5, 0.7) | ns | 0.10 (-0.50, 0.69) |
| **MLP + BioPB** | 102.7 (3.744) | 100.2 (3.473) | 99.69 (3.277) | -2.66 (-3.2, -2.1) | .0001 | -2.58 (-3.10, -2.07) | -2.94 (-3.7, -2.2) | <0.0001 | -2.84 (-3.51, -2.16) |
| **MLP + P** | 102.4 (3.132) | 99.82 (2.815) | 99.70 (3.094) | -2.74 (-3.4, -2.1) | <0.0001 | -2.66 (-3.27, -2.04) | -2.92 (-3.8, -2) | <0.0001 | -2.83 (-3.71, -1.95) |
| **Right Arm Circumference (cm)** | | | | | | | | | |
| **Control** | 34.66 (2.036) | 34.60 (2.105) | 34.87 (2.144) | -0.06 (-0.28, 0.17) | ns | -0.16 (-0.82, 0.49) | 0.21 (-0.2, 0.6) | ns | 0.62 (-0.45, 1.69) |
| **MLP + BioPB** | 34.70 (1.901) | 33.32 (1.833) | 33.19 (1.966) | -1.42 (-1.8, -1.1) | 0.0016 | -4.08 (-5.05, -3.12) | -1.56 (-2,-1.2) | <0.0001 | -4.5 (-5.65, -3.34) |
| **MLP + P** | 33.95 (1.825) | 32.88 (1.668) | 32.75 (1.655) | -1.17 (-1.5, -0.84) | 0.0004 | -3.39 (-4.33, -2.45) | -1.34 (-18, -0.9) | <0.0001 | -3.86 (-5.19, -2.53) |
| **Left Arm Circumference (cm)** | | | | | | | | | |
| **Control** | 34.55 (1.983) | 34.51 (2.035) | 34.78 (2.113) | -0.04 (-0.26, 0.17) | ns | -0.13 (-0.76, 0.51) | 0.23 (-0.1, 0.6) | ns | 0.67 (-0.34, 1.68) |
| **MLP + BioPB** | 34.60 (1.949) | 33.22 (1.896) | 33.05 (2.000) | -1.40 (-1.8, -1) | 0.0006 | -4.01 (-5.07, -2.95) | -1.59 (-2, -1.2) | <0.0001 | -4.58 (-5.69, -3.46) |
| **MLP + P** | 33.93 (1.835) | 32.92 (1.736) | 32.72 (1.770) | -1.16 (-1.5, -0.83) | 0.0003 | -3.36 (-4.29, -2.42) | -1.37 (-1.9, -0.8) | 0.0002 | -3.94 (-5.44, -2.44) |
| **Right Thigh Circumference (cm)** | | | | | | | | | |
| **Control** | 34.55 (1.983) | 34.51 (2.035) | 34.78 (2.113) | -0.04 (-0.26, 0.17) | ns | -0.13 (-0.76, 0.51) | 0.23 (-0.1, 0.6) | ns | 0.67 (-0.34, 1.68) |
| **MLP + BioPB** | 34.60 (1.949) | 33.22 (1.896) | 33.05 (2.000) | -1.40 (-1.8, -1) | <0.0001 | -4.01 (-5.07, -2.95) | -1.59 (-2, -1.2) | <0.0001 | -4.58 (-5.69, -3.46) |
| **MLP + P** | 33.93 (1.835) | 32.92 (1.736) | 32.72 (1.770) | -1.16 (-1.5, -0.83) | <0.0001 | -3.36 (-4.29, -2.42) | -1.37 (-1.9, -0.8) | <0.0001 | -3.94 (-5.44, -2.44) |
| **Left Thigh Circumference (cm)** | | | | | | | | | |
| **Control** | 55.85 (3.488) | 55.97 (3.564) | 56.14 (3.510) | 0.12 (-0.30, 0.53) | ns | 0.21 (-0.52, 0.95) | 0.29 (-0.2, 0.8) | ns | 0.51 (-0.23, 1.24) |
| **MLP + BioPB** | 55.26 (2.690) | 53.82 (2.370) | 53.32 (2.064) | -1.5 (-2.1, -1) | 0.0021 | -2.7 (-3.62, -1.84) | -1.82 (-2.5, -1.2) | <0.0001 | -3.25 (-4.29, -2.20) |
| **MLP + P** | 55.11 (2.077) | 53.49 (1.888) | 53.50 (1.878) | -1.83 (-2.4, -0.51) | 0.0016 | -3.26 (-4.26, -2.27) | -1.82(-2.5, -1.1) | <0.0001 | -3.24(-4.45, -2.03) |

|  | **Mean ± (SD)** | | | **Mean Difference from Baseline (95% CI)** | | | | | |
| --- | --- | --- | --- | --- | --- | --- | --- | --- | --- |
|  | **Week 0** | **Week 4** | **Week 8** | **Week 4** | | | **Week 8** | | |
|  |  |  |  | **Mean Difference** | ***p* value** | **Percent Difference** | **Mean Difference** | ***p* value** | **Percent Difference** |
| **Body Fat Mass (kg)** | | | | | | | | | |
| **Control** | 26.76 (4.689) | 26.94 (4.951) | 26.69 (4.686) | 0.2 (-0.6, 1.0) | ns | 0.2 (-0.6, 1.0) | -0.1 (-1.0, 0.8) | ns | -0.1 (-3.4, 3.2) |
| **MLP + BioPB** | 27.16 (5.452) | 23.04 (5.484) | 22.35 (5.514) | -4.2 (-5.3, -3.2) | 0.0004 | -16.2 (-20.7, -11.6) | -4.7 (-5.9, -3.6) | <0.0001 | **-17.9 (-22.7, -13.1)** |
| **MLP + P** | 28.00 (4.276) | 24.45 (4.055) | 24.23 (4.755) | -3.7 (-4.5, -2.8) | 0.0008 | -13.0 (-16.1, -9.9) | -4.2 (-5.7, -2.8) | 0.0002 | -15.1 (-20.6, -9.7) |
| **Percentage Body Fat (%)** | | | | | | | | | |
| **Control** | 33.28 (7.052) | 33.59 (7.078) | 33.14 (6.611) | 0.3 (-0.4, 1.1) | ns | 0.9 (-1.3, 3.2) | -0.1 (-1.0, 1.0) | ns | -0.1 (-3.4, 3.2) |
| **MLP + BioPB** | 33.48 (5.976) | 30.27 (6.691) | 29.58 (6.814) | -3.3 (-4.3, -2.3) | 0.0011 | -10.7 (-14.9, -6.6) | -3.8 (-4.9, -2.8) | <0.0001 | -12.2 (-16.2, -8.3) |
| **MLP + P** | 35.06 (4.539) | 32.49 (4.933) | 32.25 (5.260) | -2.6 (-3.4, -1.8) | 0.0016 | -7.6 (-10.2, -5.0) | -3.3 (-4.6, -1.9) | <0.0001 | -9.5 (-13.7, -5.3) |
| **Visceral fat Level** | | | | | | | | | |
| **Control** | 12.44 (2.874) | 12.75 (2.840) | 12.13 (2.802) | 0.3 (-0.2,0.8) | ns | 2.9 (-1.0, 6.8) | -0.3 (-0.9, 0.3) | ns | -2.0 (-6.4, 2.3) |
| **MLP + BioPB** | 12.61 (3.127) | 10.33 (3.049) | 10.12 (3.080) | -2.4 (-3.0,-1.7) | <0.0001 | -19.1 (-24.3, -13.8) | -2.5 (-3.1,-1.9) | <0.0001 | -20.3 (-24.7, -15.9) |
| **MLP + P** | 13.00 (2.761) | 11.12 (2.713) | 11.06 (2.955) | -1.9 (-2.4,-1.4) | 0.0008 | -14.9 (-18.8, -11.0) | -2.3 (-3.1, -1.4) | 0.0002 | -17.4 (24.4, -10.4) |
| **Skeletal Muscle Mass (kg)** | | | | | | | | | |
| **Control** | 30.86 (8.247) | 30.66 (8.244) | 30.99 (8.240) | -0.2 (-0.5, 0.1) | ns | -0.7 (-1.6, 0.3) | 0.1 (-0.1, 0.4) | ns | 0.5(-0.4, 1.4) |
| **MLP + BioPB** | 30.26 (6.051) | 29.78 (6.042) | 29.76 (5.966) | -0.5 (-0.9, -0.1) | 0.0267 | -1.6 (-2.7, -0.5) | -0.5 (-0.9, -0.1) | 0.0207 | -1.5 (-2.6, -0.4) |
| **MLP + P** | 28.85 (4.503) | 28.28 (4.510) | 28.17 (4.323) | -0.6 (-1.1, -0.2) | 0.004 | -2.2 (-3.6, -0.8) | -0.6 (-1.1, -0.1) | ns | -1.9 (-3.4, -0.4) |
| **Soft Lean Mass (kg)** | | | | | | | | | |
| **Control** | 52.08 (12.95) | 51.63 (12.79) | 52.14 (12.82) | -0.4 (-0.9, 0.0) | ns | -0.8 (-1.8, 0.1) | 0.1 (-0.4, 0.5) | ns | 0.2(-0.7, 1.1) |
| **MLP + BioPB** | 51.15 (9.489) | 50.39 (9.443) | 50.46 (9.369) | -0.8 (-1.4, -0.2) | 0.0431 | -1.5 (-2.5, -0.4) | -0.7 (-1.2, -0.1) | 0.0267 | -1.2 (-2.2, -0.3) |
| **MLP + P** | 49.04 (7.010) | 48.01 (6.906) | 47.90 (6.623) | -1.1 (-1.8 -0.4) | 0.004 | -2.2 (-3.5, -0.9) | -1.0 (-1.7, 0) | ns | -1.1 (-2.4, -1.4) |
| **Fat Free Mass (kg)** | | | | | | | | | |
| **Control** | 55.41 (13.81) | 54.90 (13.63) | 55.46 (13.67) | -0.5 (-1.0, 0.0) | ns | -0.9 (-1.9, 0.1) | 0.0 (-0.4, 0.5) | ns | 0.2 (-0.7, 1.0) |
| **MLP + BioPB** | 54.41 (10.14) | 53.61 (10.10) | 53.71 (10.02) | -0.8 (-1.5, -0.2) | 0.034 | -1.5 (-2.5, -0.4) | -0.7 (-1.3, -0.1) | 0.034 | -1.2 (-2.1, -0.2) |
| **MLP + P** | 52.13 (7.426) | 51.05 (7.300) | 50.93 (6.995) | -1.1 (-1.9, -0.4) | 0.0029 | -2.1 (-3.4, -0.9) | -1.0 (-1.8, -0.2) | ns | -1.8 (-3.3, -0.4) |
| **Total Body Water (L)** | | | | | | | | | |
| **Control** | 40.54 (10.03) | 40.15 (9.881) | 40.54 (9.918) | -0.4 (-0.8, 0.0) | ns | -0.9 (-1.9, 0.1) | 0.0 (-0.3, 0.3) | ns | 0.1 (-0.8, 1.0) |
| **MLP + BioPB** | 39.80 (7.362) | 39.22 (7.342) | 39.29 (7.278) | -0.6 (-1.1, -0.1) | 0.016 | -1.5 (-2.5,-0.4) | -0.5 (-0.9, -0.1) | 0.016 | -1.2 (-2.1, -0.2) |
| **MLP + P** | 38.12 (5.295) | 37.36 (5.320) | 37.29 (5.128) | -0.8 (-1.4, -0.3) | 0.004 | -2.2 (-3.5, -0.9) | -0.7 (-1.3, -0.1) | ns | -1.8 (-3.3, -0.4) |

**Table S2.** Clinical biochemistry indices at Weeks 0 (preintervention), 4 (post-intervention) and 8 (4 weeks after the intervention had ceased). Trial details are described in Materials and Methods and in the Figure S1 (Supplementary table 1) legend. Data represents the mean ± SD at baseline (Week 0), after the intervention (Week 4) and 4 weeks postintervention (Week 8), the mean differences (Δ) with the 95% CI from baseline and *p* values at Week 4 and Week 8.

|  | **Mean ± (SD)** | | | **Mean Difference from Baseline (95% CI)** | | | |
| --- | --- | --- | --- | --- | --- | --- | --- |
|  | **Week 0** | **Week 4** | **Week 8** | **Week 4** | | **Week 8** | |
|  |  |  |  | **Mean Difference** | ***p* value** | **Mean Difference** | ***p* value** |
| **Creatinine (μmol/L)** | | | | | | | |
| **Control** | 66.79 (15.38) | 67.71 (15.10) | 67.5 (9.59) | 0.9286  (-1.803, 3.660) | ns | 0.7143  (-1.622, 3.051 | ns |
| **MLP + BioPB** | 72.94 (9.59) | 72.18 (8.16) | 68.24 (9.48) | -0.7647  (-3.831, 2.302) | ns | -4.706  (-7.832, -1.580 | 0.0157 |
| **MLP + P** | 68.00 (10.85) | 68.69 (13.69) | 64.56 (11.73) | 0.6875  (-2.014, 3.389 | ns | -3.438  (-5.929, -0.9460) | 0.0160 |
| **Bilirubin (total; μmol/L)** | | | | | | | |
| **Control** | 5.93 (2.70) | 6.21 (1.30) | 6.36 (2.81) | -0.3333  (-2.337, 1.671 | ns | 0.8667  (-1.016, 2.749 | ns |
| **MLP + BioPB** | 8.53 (4.87) | 8.65 (2.96) | 10.41 (6.68) | 0.1176  (-1.369, 1.604 | ns | 1.882  (-0.2829, 4.048 | ns |
| **MLP + P** | 5.47 (2.54) | 6.13 (2.44) | 6.47 (2.00) | 1.063  (-0.6937, 2.819) | ns | 0.4375  (-1.236, 2.111 | ns |
| **ALP (units/L)** | | | | | | | |
| **Control** | 75.14 (26.24) | 73.79 (19.49) | 74.07 (24.09) | -1.357-6.949  4.234 | ns | -1.071-5.581  3.438 | ns |
| **MLP + BioPB** | 63.29 (14.71) | 54.82 (11.67) | 60.00 (13.39) | -8.471-11.86  -5.079 | 0.0001 | -3.294-6.684  0.09580 | ns |
| **MLP + P** | 67.63 (16.97) | 60.31 (17.37) | 68.50 (15.69) | -7.313-12.40  -2.229 | 0.0094 | 0.8750-3.712  5.462 | ns |
| **GGT (units/L)** | | | | | | | |
| **Control** | 12.36 (7.05) | 11.71 (5.38) | 12.00 (7.71) | -0.6429  (-2.013, 0.7274 | ns | -0.3571  (-1.503, 0.7889 | ns |
| **MLP + BioPB** | 15.44 (20.63) | 9.94 (7.72) | 12.00 (12.35) | -9.647  (-20.46, 1.168 | 0.0071 | -7.176  (-16.88, 2.527 | ns |
| **MLP + P** | 11.38 (12.15) | 8.81 (9.54) | 11.06 (13.48) | -2.563  (-4.594, -0.5314) | ns | -0.3125  (-3.478, 2.853 | ns |
| **ALT (units/L)** | | | | | | | |
| **Control** | 27.29 (12.72) | 22.71 (8.70) | 23.38 (8.70) | -4.571  (-7.530, -1.612) | 0.0050 | -3.429  (-7.141, 0.2838) | ns |
| **MLP + BioPB** | 27.35 (14.19) | 25.47 (7.62) | 23.12 (9.78) | -1.882  (-6.685, 2.920) | ns | -4.235  (-7.869, -0.6018) | ns |
| **MLP + P** | 20.81 (5.37) | 21.13 (6.37) | 23.75 (9.33) | 0.3125  (-2.883, 3.508 | ns | 2.938  (-1.758, 7.633) | ns |
| **AST (units/L)** | | | | | | | |
| **Control** | 27.36 (6.79) | 23.43 (2.52) | 22.29 (4.06) | -3.929  (-5.660, -2.197) | 0.0019 | -5.071  (-7.128, -3.015) | 0.0003 |
| **MLP + BioPB** | 25.81 (5.45) | 22.94 (5.14) | 20.88 (4.25) | -1.235  (-5.653, 3.182) | ns | -5.059  (-7.167, -2.951) | 0.0009 |
| **MLP + P** | 23.31 (4.08) | 20.13 (6.51) | 20.56 (4.80) | -3.188  (-5.892, -0.4829) | 0.0160 | -2.750  (-4.474, -1.026) | 0.0340 |
| **Protein (total; g/L)** | | | | | | | |
| **Control** | 68.86 (4.97) | 69.21 (4.72) | 69.71 (6.39) | 0.3571  (-1.222, 1.936) | ns | 0.8571  (-1.217, 2.931) | ns |
| **MLP + BioPB** | 69.82 (3.33) | 68.82 (4.27) | 68.24 (4.59) | -1.000  (-2.443, 0.4428) | ns | -1.588  (-2.949, -0.2272) | ns |
| **MLP + P** | 69.25 (3.99) | 67.63 (2.30) | 69.56 (3.71) | -1.625  (-2.999, -0.2509) | ns | 0.3125  (-0.8825, 1.507) | ns |
| **Albumin (g/L)** | | | | | | | |
| **Control** | 39.64 (1.78) | 39.43 (1.51) | 39.57 (2.74) | -0.2143  (-1.305, 0.8760) | ns | -0.0714  (-1.362, 1.219) | ns |
| **MLP + BioPB** | 41.76 (2.24) | 41.65 (1.99) | 40.41 (2.66) | -0.1176  (-1.176, 0.9405) | ns | -1.353  (-2.296, -0.4094) | ns |
| **MLP + P** | 40.81 (2.04) | 40.19 (2.04) | 39.44 (2.92) | -0.6250  (-1.375, 0.1254) | ns | -1.375  (-2.474, -0.2765) | ns |
| **Globulin (g/L)** | | | | | | | |
| **Control** | 29.50 (3.94) | 29.79 (3.53) | 30.29 (4.47) | 0.2857  (-0.5745, 1.146) | ns | 0.7857  (-0.2808, 1.852) | ns |
| **MLP + BioPB** | 28.24 (2.72) | 26.82 (3.10) | 28.06 (2.98) | -1.412  (-2.266, -0.5580) | 0.0157 | -0.1765  (-1.125, 0.7722) | ns |
| **MLP + P** | 28.69 (3.09) | 27.56 (2.07) | 30.00 (2.93) | -1.125  (-2.035, -0.2150) | ns | 1.3130  (0.5660, 2.059) | 0.0267 |

**Table S3. Changes in blood pressure and pulse. Results represent mean ± SD.**

|  | **Control** | | | **MLP + BioPB** | | | **MLP + P** | | |
| --- | --- | --- | --- | --- | --- | --- | --- | --- | --- |
|  | **Week 0** | **Week 4** | **Week 8** | **Week 0** | **Week 4** | **Week 8** | **Week 0** | **Week 4** | **Week 8** |
| Systolic (mmHg) | 133.8  ± 13.49 | 132.6  ± 11.18 | 129.3  ± 10.90 | 129.6  ± 14.37 | 126.9  ± 10.73 | 128.1  ± 13.02 | 133.1  ± 17.09 | 133.2  ± 14.82 | 128.9  ± 15.77 |
| Diastolic (mmHg) | 79.50  ± 8.230 | 79.75  ± 9.815 | 76.13  ± 8.382 | 74.56  ± 11.04 | 73.39  ± 8.445 | 72.12  ± 10.32 | 75.06  ± 10.64 | 78.65  ± 9.347 | 75.25  ± 13.43 |
| Pulse (bpm) | 71.25  ± 9.630 | 72.31  ± 11.22 | 75.25  ± 12.44 | 71.83  ± 8.887 | 73.39  ± 11.73 | 72.53  ± 12.68 | 74.29  ± 11.15 | 81.00  ± 15.44 | 76.94  ± 12.11 |

**Table S4. Frequency of nausea, expressed as a percentage, reported by each group at baseline (W0), during the intervention period (week 1, 2, 3 and 4 [W1-4]), immediately after the end of the intervention (W5), and at the end of the 4-weel follow-up period (W8).**

|  | **Control** | | | | | | | **MLP + BioPB** | | | | | | | **MLP + P** | | | | | | |
| --- | --- | --- | --- | --- | --- | --- | --- | --- | --- | --- | --- | --- | --- | --- | --- | --- | --- | --- | --- | --- | --- |
|  | **W0** | **W1** | **W2** | **W3** | **W4** | **W5** | **W8** | **W0** | **W1** | **W2** | **W3** | **W4** | **W5** | **W8** | **W0** | **W1** | **W2** | **W3** | **W4** | **W5** | **W8** |
| **Nausea** | 3 | 2 | 1 | 2 | 2 | 0 | 0 | 2 | 3 | 2 | 3 | 3 | 1 | 2 | 2 | 1 | 0 | 0 | 0 | 0 | 1 |
| **Severe** | 0 | 0 | 0 | 0 | 0 | 0 | 0 | 0 | 0 | 0 | 0 | 0 | 0 | 0 | 0 | 0 | 0 | 0 | 0 | 0 | 0 |
| **Frequent** | 0 | 0 | 0 | 0 | 0 | 0 | 0 | 0 | 0 | 0 | 0 | 0 | 0 | 0 | 0 | 0 | 0 | 0 | 0 | 0 | 0 |
| **Occasional** | 0 | 0 | 0 | 0 | 0 | 0 | 0 | 0 | 0 | 0 | 0 | 0 | 0 | 0 | 0 | 0 | 0 | 0 | 0 | 0 | 0 |
| **Slight** | 0 | 0 | 0 | 0 | 0 | 0 | 0 | 0 | 1 | 0 | 0 | 0 | 0 | 0 | 0 | 1 | 0 | 0 | 0 | 0 | 0 |
| **Minimal** | 3 | 2 | 1 | 2 | 2 | 0 | 0 | 2 | 2 | 2 | 3 | 3 | 1 | 2 | 2 | 0 | 0 | 0 | 0 | 0 | 1 |
| **None** | 13 | 13 | 14 | 14 | 14 | 15 | 15 | 16 | 14 | 16 | 15 | 15 | 17 | 16 | 14 | 14 | 16 | 16 | 16 | 16 | 14 |
| ***n*** | 16 | 15 | 15 | 16 | 16 | 15 | 15 | 18 | 17 | 18 | 18 | 18 | 18 | 18 | 16 | 15 | 16 | 16 | 16 | 16 | 15 |

**Table S5. Frequency of stomach pain, expressed as a percentage, reported by each group at baseline (W0), during the intervention period (week 1, 2, 3 and 4 [W1-4]), immediately after the end of the intervention (W5), and at the end of the 4-week follow-up period (W8).**

|  | **Control** | | | | | | | **MLP + BioPB** | | | | | | | **MLP + P** | | | | | | |
| --- | --- | --- | --- | --- | --- | --- | --- | --- | --- | --- | --- | --- | --- | --- | --- | --- | --- | --- | --- | --- | --- |
|  | **W0** | **W1** | **W2** | **W3** | **W4** | **W5** | **W8** | **W0** | **W1** | **W2** | **W3** | **W4** | **W5** | **W8** | **W0** | **W1** | **W2** | **W3** | **W4** | **W5** | **W8** |
| **Stomach pain** | 2 | 4 | 2 | 2 | 2 | 1 | 2 | 6 | 2 | 3 | 1 | 4 | 3 | 3 | 4 | 2 | 3 | 2 | 3 | 2 | 1 |
| **Severe** | 0 | 0 | 0 | 0 | 0 | 0 | 0 | 0 | 0 | 0 | 0 | 0 | 0 | 0 | 0 | 0 | 0 | 0 | 0 | 0 | 0 |
| **Frequent** | 0 | 0 | 0 | 0 | 0 | 0 | 0 | 0 | 0 | 0 | 0 | 0 | 0 | 0 | 0 | 0 | 0 | 0 | 0 | 0 | 0 |
| **Occasional** | 0 | 0 | 0 | 0 | 0 | 0 | 0 | 0 | 0 | 0 | 0 | 0 | 0 | 0 | 0 | 0 | 0 | 0 | 0 | 0 | 0 |
| **Slight** | 0 | 0 | 0 | 1 | 1 | 0 | 1 | 1 | 0 | 0 | 0 | 0 | 0 | 0 | 0 | 1 | 0 | 0 | 0 | 0 | 0 |
| **Minimal** | 2 | 4 | 2 | 1 | 1 | 1 | 1 | 5 | 2 | 3 | 1 | 4 | 3 | 3 | 4 | 1 | 3 | 2 | 3 | 2 | 1 |
| **None** | 14 | 11 | 13 | 14 | 14 | 14 | 13 | 12 | 15 | 15 | 17 | 14 | 15 | 15 | 12 | 13 | 13 | 14 | 13 | 14 | 14 |
| ***n*** | 16 | 15 | 15 | 16 | 16 | 15 | 15 | 18 | 17 | 18 | 18 | 18 | 18 | 18 | 16 | 15 | 16 | 16 | 16 | 16 | 15 |

**Table S6. Frequency of abdominal discomfort/cramps, expressed as a percentage, reported by each group at baseline (W0), during the intervention period (week 1, 2, 3 and 4 [W1-4]), immediately after the end of the intervention (W5), and at the end of the 4-weel follow-up period (W8).**

|  | **Control** | | | | | | | **MLP + BioPB** | | | | | | | **MLP + P** | | | | | | |
| --- | --- | --- | --- | --- | --- | --- | --- | --- | --- | --- | --- | --- | --- | --- | --- | --- | --- | --- | --- | --- | --- |
|  | **W0** | **W1** | **W2** | **W3** | **W4** | **W5** | **W8** | **W0** | **W1** | **W2** | **W3** | **W4** | **W5** | **W8** | **W0** | **W1** | **W2** | **W3** | **W4** | **W5** | **W8** |
| **Abdominal discomfort/ cramps** | 5 | 3 | 5 | 4 | 3 | 2 | 3 | 5 | 3 | 2 | 2 | 5 | 3 | 4 | 6 | 4 | 4 | 2 | 3 | 1 | 3 |
| **Severe** | 0 | 0 | 0 | 0 | 0 | 0 | 0 | 0 | 0 | 0 | 0 | 0 | 0 | 0 | 0 | 0 | 0 | 0 | 0 | 0 | 0 |
| **Frequent** | 0 | 0 | 0 | 0 | 0 | 0 | 0 | 0 | 0 | 0 | 0 | 0 | 0 | 0 | 0 | 0 | 0 | 0 | 0 | 0 | 0 |
| **Occasional** | 0 | 0 | 0 | 0 | 0 | 0 | 0 | 0 | 0 | 0 | 0 | 0 | 0 | 0 | 0 | 0 | 0 | 0 | 0 | 0 | 0 |
| **Slight** | 0 | 0 | 1 | 0 | 1 | 0 | 1 | 1 | 0 | 0 | 0 | 0 | 0 | 0 | 1 | 1 | 0 | 0 | 0 | 0 | 0 |
| **Minimal** | 5 | 3 | 4 | 4 | 2 | 2 | 2 | 4 | 3 | 2 | 2 | 5 | 3 | 4 | 5 | 3 | 4 | 2 | 3 | 1 | 3 |
| **None** | 11 | 12 | 10 | 12 | 13 | 13 | 12 | 13 | 14 | 16 | 16 | 13 | 15 | 14 | 10 | 11 | 12 | 14 | 13 | 15 | 12 |
| ***n*** | 16 | 15 | 15 | 16 | 16 | 15 | 15 | 18 | 17 | 18 | 18 | 18 | 18 | 18 | 16 | 15 | 16 | 16 | 16 | 16 | 15 |

**Table S7. Frequency of bloating, expressed as a percentage, reported by each group at baseline (W0), during the intervention period (weeks 1, 2, 3 and 4 [W1-4]), immediately after the end of the intervention (W5), and at the end of the 4-weel follow-up period (W8).**

|  | **Control** | | | | | | | **MLP + BioPB** | | | | | | | **MLP + P** | | | | | | |
| --- | --- | --- | --- | --- | --- | --- | --- | --- | --- | --- | --- | --- | --- | --- | --- | --- | --- | --- | --- | --- | --- |
|  | **W0** | **W1** | **W2** | **W3** | **W4** | **W5** | **W8** | **W0** | **W1** | **W2** | **W3** | **W4** | **W5** | **W8** | **W0** | **W1** | **W2** | **W3** | **W4** | **W5** | **W8** |
| **Bloating** | 10 | 4 | 6 | 6 | 4 | 5 | 5 | 14 | 8 | 6 | 6 | 10 | 5 | 6 | 11 | 5 | 4 | 5 | 4 | 4 | 4 |
| **Severe** | 0 | 0 | 0 | 0 | 0 | 0 | 0 | 0 | 0 | 0 | 0 | 0 | 0 | 0 | 0 | 0 | 0 | 0 | 0 | 0 | 0 |
| **Frequent** | 0 | 0 | 0 | 0 | 0 | 0 | 0 | 2 | 0 | 0 | 0 | 0 | 0 | 0 | 0 | 1 | 0 | 0 | 0 | 0 | 0 |
| **Occasional** | 2 | 0 | 0 | 1 | 1 | 0 | 1 | 2 | 0 | 0 | 0 | 0 | 0 | 0 | 0 | 0 | 0 | 0 | 0 | 0 | 0 |
| **Slight** | 3 | 1 | 0 | 0 | 0 | 0 | 1 | 2 | 1 | 2 | 1 | 1 | 0 | 0 | 4 | 0 | 1 | 2 | 1 | 0 | 0 |
| **Minimal** | 5 | 3 | 6 | 5 | 3 | 5 | 3 | 8 | 7 | 4 | 5 | 9 | 5 | 6 | 7 | 4 | 3 | 3 | 3 | 4 | 4 |
| **None** | 6 | 11 | 9 | 10 | 12 | 10 | 10 | 4 | 9 | 12 | 12 | 8 | 13 | 12 | 5 | 10 | 12 | 11 | 12 | 12 | 11 |
| ***n*** | 16 | 15 | 15 | 16 | 16 | 15 | 15 | 18 | 17 | 18 | 18 | 18 | 18 | 18 | 16 | 15 | 16 | 16 | 16 | 16 | 15 |

**Table S8. Frequency of diarrhoea, expressed as a percentage, reported by each group at baseline (W0), during the intervention period (week 1, 2, 3 and 4 [W1-4]), immediately after the end of the intervention (W5), and at the end of the 4-weel follow-up period (W8).**

|  | **Control** | | | | | | | **MLP + BioPB** | | | | | | | **MLP + P** | | | | | | |
| --- | --- | --- | --- | --- | --- | --- | --- | --- | --- | --- | --- | --- | --- | --- | --- | --- | --- | --- | --- | --- | --- |
|  | **W0** | **W1** | **W2** | **W3** | **W4** | **W5** | **W8** | **W0** | **W1** | **W2** | **W3** | **W4** | **W5** | **W8** | **W0** | **W1** | **W2** | **W3** | **W4** | **W5** | **W8** |
| **Diarrhoea** | 4 | 2 | 3 | 2 | 2 | 2 | 1 | 8 | 4 | 5 | 2 | 3 | 2 | 3 | 2 | 1 | 1 | 2 | 3 | 2 | 3 |
| **Severe** | 0 | 0 | 0 | 0 | 0 | 0 | 0 | 0 | 0 | 0 | 0 | 0 | 0 | 0 | 0 | 0 | 0 | 0 | 0 | 0 | 0 |
| **Frequent** | 0 | 0 | 0 | 0 | 0 | 0 | 0 | 0 | 0 | 0 | 0 | 0 | 0 | 0 | 0 | 0 | 0 | 0 | 0 | 0 | 0 |
| **Occasional** | 1 | 0 | 0 | 0 | 0 | 0 | 0 | 2 | 0 | 0 | 0 | 0 | 0 | 0 | 0 | 0 | 0 | 0 | 0 | 0 | 0 |
| **Slight** | 0 | 1 | 0 | 0 | 0 | 0 | 0 | 0 | 0 | 2 | 0 | 0 | 1 | 0 | 0 | 0 | 0 | 0 | 1 | 0 | 0 |
| **Minimal** | 3 | 1 | 3 | 2 | 2 | 2 | 1 | 6 | 4 | 3 | 2 | 3 | 1 | 3 | 2 | 1 | 1 | 2 | 2 | 2 | 3 |
| **None** | 12 | 13 | 12 | 14 | 14 | 13 | 14 | 10 | 13 | 13 | 16 | 15 | 16 | 15 | 14 | 14 | 15 | 14 | 13 | 14 | 12 |
| ***n*** | 16 | 15 | 15 | 16 | 16 | 15 | 15 | 18 | 17 | 18 | 18 | 18 | 18 | 18 | 16 | 15 | 16 | 16 | 16 | 16 | 15 |

**Table S9. Frequency of flatulence, expressed as a percentage, reported by each group at baseline (W0), during the intervention period (week 1, 2, 3 and 4 [W1-4]), immediately after the end of the intervention (W5), and at the end of the 4-weel follow-up period (W8).**

|  | **Control** | | | | | | | **MLP + BioPB** | | | | | | | **MLP + P** | | | | | | |
| --- | --- | --- | --- | --- | --- | --- | --- | --- | --- | --- | --- | --- | --- | --- | --- | --- | --- | --- | --- | --- | --- |
|  | **W0** | **W1** | **W2** | **W3** | **W4** | **W5** | **W8** | **W0** | **W1** | **W2** | **W3** | **W4** | **W5** | **W8** | **W0** | **W1** | **W2** | **W3** | **W4** | **W5** | **W8** |
| **Flatulence** | 14 | 9 | 10 | 9 | 11 | 10 | 9 | 16 | 8 | 9 | 8 | 10 | 9 | 11 | 10 | 5 | 5 | 5 | 6 | 5 | 7 |
| **Severe** | 1 | 1 | 0 | 0 | 1 | 0 | 0 | 0 | 0 | 0 | 0 | 0 | 0 | 0 | 1 | 0 | 0 | 0 | 0 | 0 | 0 |
| **Frequent** | 0 | 0 | 0 | 1 | 0 | 1 | 0 | 2 | 0 | 0 | 1 | 1 | 1 | 1 | 1 | 0 | 1 | 0 | 0 | 0 | 0 |
| **Occasional** | 2 | 2 | 1 | 1 | 2 | 1 | 2 | 2 | 0 | 2 | 0 | 1 | 1 | 0 | 1 | 2 | 2 | 2 | 1 | 0 | 0 |
| **Slight** | 3 | 2 | 2 | 2 | 1 | 1 | 3 | 1 | 2 | 0 | 0 | 0 | 0 | 5 | 2 | 0 | 1 | 0 | 3 | 2 | 3 |
| **Minimal** | 8 | 4 | 7 | 5 | 7 | 7 | 4 | 11 | 6 | 7 | 7 | 8 | 7 | 5 | 5 | 3 | 1 | 3 | 2 | 3 | 4 |
| **None** | 2 | 6 | 5 | 7 | 5 | 5 | 6 | 2 | 9 | 9 | 10 | 8 | 9 | 7 | 6 | 10 | 11 | 11 | 10 | 11 | 8 |
| ***n*** | 16 | 15 | 15 | 16 | 16 | 15 | 15 | 18 | 17 | 18 | 18 | 18 | 18 | 18 | 16 | 15 | 16 | 16 | 16 | 16 | 15 |

**Table S10. Frequency of constipation, expressed as a percentage, reported by each group at baseline (W0), during the intervention period (week 1, 2, 3 and 4 [W1-4]), immediately after the end of the intervention (W5), and at the end of the 4-weel follow-up period (W8).**

|  | **Control** | | | | | | | **MLP + BioPB** | | | | | | | **MLP + P** | | | | | | |
| --- | --- | --- | --- | --- | --- | --- | --- | --- | --- | --- | --- | --- | --- | --- | --- | --- | --- | --- | --- | --- | --- |
|  | **W0** | **W1** | **W2** | **W3** | **W4** | **W5** | **W8** | **W0** | **W1** | **W2** | **W3** | **W4** | **W5** | **W8** | **W0** | **W1** | **W2** | **W3** | **W4** | **W5** | **W8** |
| **Constipation** | 2 | 4 | 6 | 5 | 5 | 5 | 2 | 9 | 8 | 11 | 8 | 9 | 9 | 8 | 9 | 7 | 8 | 9 | 9 | 8 | 6 |
| **Severe** | 0 | 0 | 0 | 0 | 0 | 0 | 0 | 0 | 0 | 0 | 0 | 0 | 0 | 0 | 0 | 1 | 1 | 1 | 0 | 0 | 0 |
| **Frequent** | 0 | 0 | 0 | 0 | 1 | 0 | 0 | 0 | 0 | 1 | 0 | 0 | 0 | 0 | 1 | 1 | 1 | 1 | 1 | 0 | 0 |
| **Occasional** | 1 | 0 | 0 | 0 | 0 | 0 | 1 | 2 | 0 | 0 | 0 | 1 | 0 | 0 | 1 | 1 | 0 | 4 | 0 | 0 | 0 |
| **Slight** | 1 | 0 | 3 | 3 | 0 | 2 | 0 | 4 | 2 | 0 | 3 | 2 | 2 | 1 | 4 | 1 | 3 | 0 | 4 | 2 | 1 |
| **Minimal** | 0 | 4 | 3 | 2 | 4 | 3 | 1 | 3 | 6 | 10 | 5 | 6 | 7 | 7 | 3 | 3 | 3 | 3 | 4 | 6 | 5 |
| **None** | 14 | 11 | 9 | 11 | 11 | 10 | 13 | 9 | 9 | 7 | 10 | 9 | 9 | 10 | 7 | 8 | 8 | 7 | 7 | 8 | 9 |
| ***n*** | 16 | 15 | 15 | 16 | 16 | 15 | 15 | 18 | 17 | 18 | 18 | 18 | 18 | 18 | 16 | 15 | 16 | 16 | 16 | 16 | 15 |

**Reference**

1. Knechtle B, Tanous DR, Wirnitzer G, Leitzmann C, Rosemann T, Scheer V, Wirnitzer K. Training and Racing Behavior of Recreational Runners by Race Distance—Results From the NURMI Study (Step 1). Frontiers in physiology. 2021;12:620404-. doi: 10.3389/fphys.2021.620404.

2. Murtagh EM, Mair JL, Aguiar E, Tudor-Locke C, Murphy MH. Outdoor Walking Speeds of Apparently Healthy Adults: A Systematic Review and Meta-analysis. Sports medicine (Auckland). 2021;51(1):125-41. doi: 10.1007/s40279-020-01351-3.

3. Gupta S, Stanula A, Goswami A, Adhikari A, Singh A, Ostrowski A. Relationship between cycling speed and blood lactate level at various intervals following 1-km time trial cycling. Antropomotoryka. 2022;32(97):29-36. doi: 10.5604/01.3001.0015.8589.

4. Bassett DR, Toth LP, LaMunion SR, Crouter SE. Step Counting: A Review of Measurement Considerations and Health-Related Applications. Sports medicine (Auckland). 2017;47(7):1303-15. doi: 10.1007/s40279-016-0663-1.
